# Co-production of high-purity floridoside and isofloridoside ameliorates MASH via *Parabacteroides goldsteinii*-UDCA-FXR enterohepatic axis

**DOI:** 10.1186/s13020-026-01450-9

**Published:** 2026-07-17

**Authors:** Shuang Li, Rong Guo, Linxiao Sun, Peng Zhu, Tingting Wang, JianJun Zheng, Haimin Chen, Hongshan Li

**Affiliations:** 1 Ningbo No.2 Hospital, Wenzhou Medical University, Ningbo, 315000 China; 2 Hangzhou Medical College, Hangzhou, 310053 Zhejiang China; 3 Collaborative Innovation Center for Zhejiang Marine High‑Efficiency and Healthy Aquaculture, Ningbo University, Ningbo, 315211 Zhejiang China

**Keywords:** Co-production of Floridoside and Isofloridoside, *Pyropia haitanensis*, Metabolic dysfunction-associated steatohepatitis, *Parabacteroides goldsteinii*, Gut-liver axis, Alternative bile acid synthesis pathway

## Abstract

**Background:**

Metabolic dysfunction-associated steatohepatitis (MASH), the progressive form of metabolic dysfunction-associated fatty liver disease (MAFLD), is tightly linked to gut microbiota dysbiosis and disrupted bile acid (BA) homeostasis. Floridoside (Flor), a marine glycoside from the edible seaweed *Pyropia haitanensis* (*P. haitanensis*), exerts promising biological activities. However, protocols for its high-purity preparation and the mechanisms underlying its anti-MASH effects remain unclear.

**Purpose:**

To develop a protocol for the preparation of high-purity Flor and its isomer isofloridoside (Isoflor) from *P. haitanensis*, and to elucidate how Flor alleviates MASH via regulating gut microbiota and BA metabolism.

**Methods:**

High-purity Flor and Isoflor were isolated via integrated chromatography, with their chemical structures confirmed by LC–MS and NMR. Anti-MASH efficacy was evaluated in a high-fat diet (HFD)-induced murine MASH model. The underlying mechanisms were explored using multi-omics analyses, including transcriptomics, gut microbiota metagenomics and BA-targeted metabolomics, and further validated by molecular docking, molecular dynamics simulation and western blotting; the compounds’ biosafety was evaluated using zebrafish.

**Results:**

High-purity Flor and Isoflor were successfully isolated, each with a purity of ≥ 99.0%. Both compounds exhibited a favorable biosafety profile and comparable lipid-lowering activity in zebrafish. In HFD-induced murine MASH models, Flor robustly ameliorated HFD-driven obesity, hepatic steatosis, and chronic inflammation, and restored systemic BA homeostasis characterized by a markedly increased non-12-OH/12-OH BA ratio. Meanwhile, Flor treatment dramatically enriched the relative abundance of intestinal *Parabacteroides goldsteinii* (*P. goldsteinii*), which showed a significant positive correlation with MASH alleviation and beneficial BAs (e.g., ursodeoxycholic acid (UDCA)). Mechanistically, UDCA exerted its therapeutic effects by antagonizing FXR signaling, upregulating the hepatic protein and mRNA expression of CYP7B1 and CYP27A1, and ultimately promoting the activation of the alternative BA synthesis pathway.

**Conclusion:**

High-purity Flor and Isoflor were obtained via an integrated co-production process from *P. haitanensis*. We hypothesize that Flor may ameliorate MASH by enriching *P. goldsteinii* and modulating the UDCA-FXR axis to activate the alternative bile acid synthesis pathway, positioning Flor as a promising prebiotic candidate for MASH management.

**Graphical Abstract:**

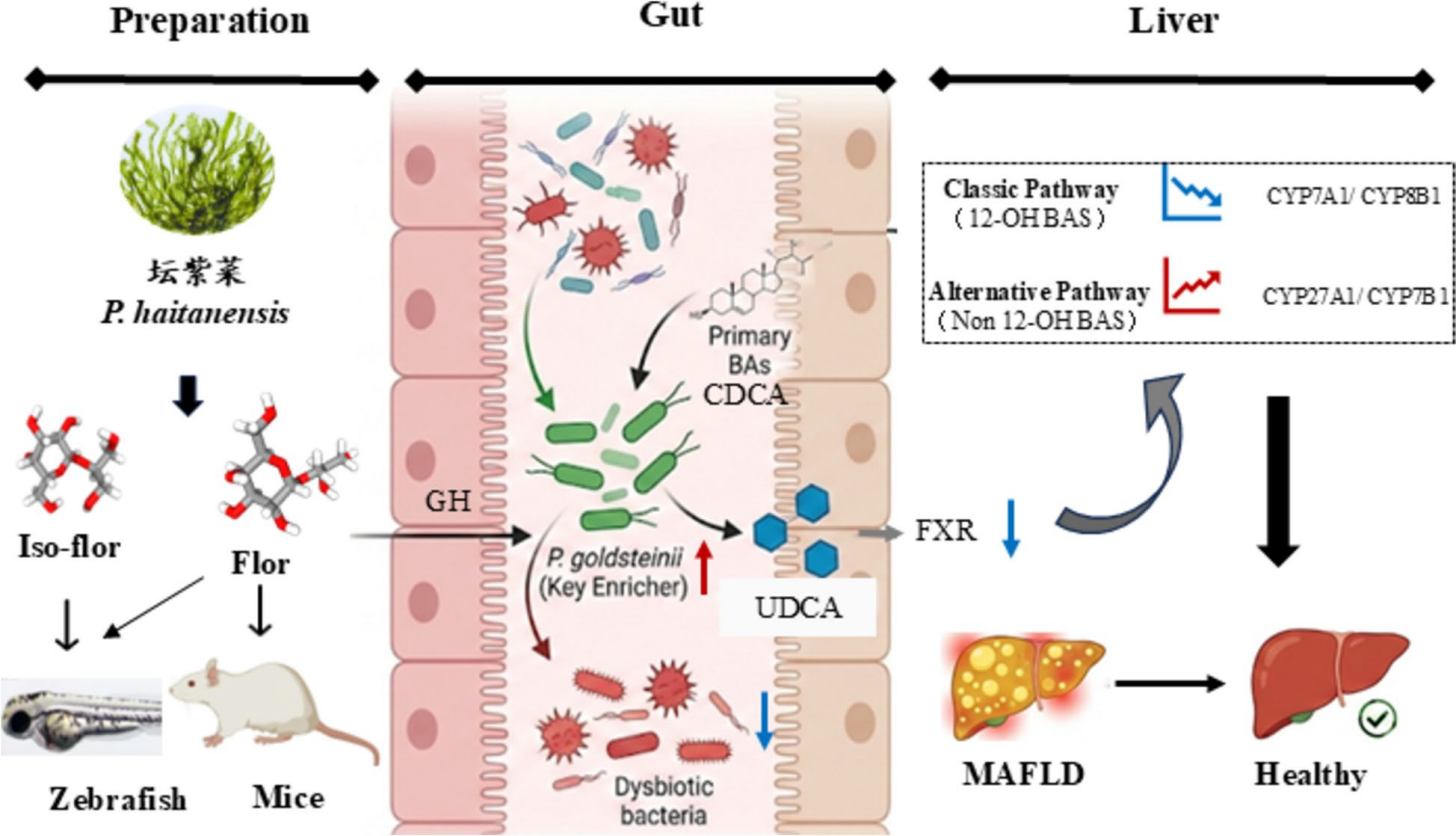

**Supplementary Information:**

The online version contains supplementary material available at 10.1186/s13020-026-01450-9.

## Introduction

Metabolic dysfunction-associated steatohepatitis (MASH), the progressive subtype of metabolic dysfunction-associated fatty liver disease (MAFLD), is manifested by hepatic steatosis, lobular inflammation and fibrosis, and substantially increases the risk of cirrhosis and hepatocellular carcinoma (HCC) [21]. With its global prevalence rising rapidly, there are currently no approved first-line drugs for MASH. Leading investigational candidates such as the farnesoid X receptor (FXR) agonist obeticholic acid (OCA) suffer from variable clinical efficacy and dose-related adverse reactions [22, 26]. This underscores an urgent demand for effective and low-toxic natural products as alternative interventions for MASH.

Dysregulated bile acid (BA) metabolism and gut microbiota dysbiosis are well-recognized core pathogenic drivers of MASH progression, and the two pathways are closely linked through the gut-liver axis. FXR serves as the key nuclear receptor regulating enterohepatic BA circulation and whole-body metabolic homeostasis [1, 3]. Hepatic BA synthesis proceeds via two evolutionarily conserved pathways. The classic pathway predominantly generates 12α-hydroxylated BAs (12-OH BAs), which are strongly correlated with hepatic steatosis and inflammation. In contrast, the alternative pathway yields hydrophilic and metabolically favorable non-12α-hydroxylated BAs (non-12-OH BAs), whose synthesis relies on the rate-limiting enzymes CYP7B1 and CYP27A1 [7]. Importantly, activated FXR strongly represses the transcription of CYP7B1 and CYP27A1, which restricts the flux of the alternative BA synthesis pathway and reduces the production of protective non-12-OH BAs [4, 24]. Growing evidence from clinical cohorts and preclinical mechanistic studies confirms that non-12-OH BAs exert prominent protective effects against metabolic liver disorders by functioning as endogenous selective FXR modulators to regulate hepatic BA metabolism and sustain metabolic homeostasis [8, 10, 11]. Since these beneficial non-12-OH BAs are mainly produced by the CYP7B1-dependent alternative BA synthesis pathway, we hypothesize that targeted modulation of this pathway via precise regulation of FXR signaling may provide a promising and safe dietary strategy for MASH prevention and treatment.

Floridosides are typical low-molecular-weight glycosides abundant in the edible red seaweed *Pyropia haitanensis* (*P. haitanensis*), making up 2.5%–10.8% of the algal dry weight [2, 20, 30]. They share a common core structure composed of a galactose moiety bound to glycerol, with a molecular weight of 252 Da [6, 19]. Naturally existing as two major stereoisomers, floridoside (Flor, α-D-galactopyranosyl-(1 → 2)-D-glycerol) and isofloridoside (Isoflor, α-D-galactopyranosyl-(1 → 1)-D/L-glycerol), both molecules possess diverse biological functions, including immunomodulation [9], antibacterial activity [25] and antioxidant capacity [14]. Previous studies have demonstrated that dietary intake of *P. haitanensis* can regulate gut microbiota and reduce MASH risk, with glycosides recognized as its major bioactive components [23, 28, 29]. Nevertheless, high-purity monomeric Flor and Isoflor are not yet commercially available, owing to their high structural similarity and strong water solubility [5, 17, 27]. Such technical limitations have greatly impeded further research on their structure–activity relationships and pharmacological mechanisms, especially regarding their roles in MASH.

**Fig. 1:**
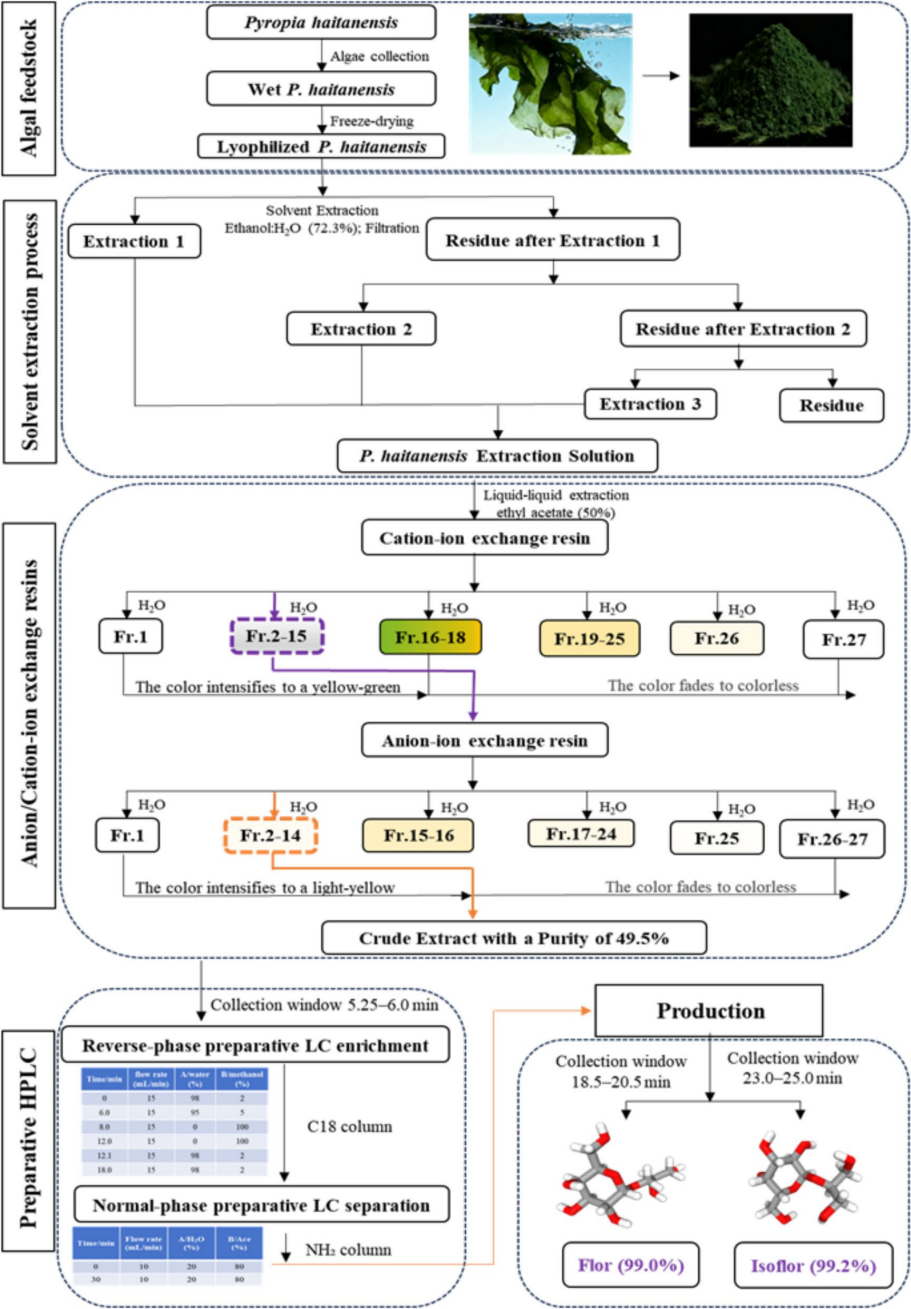
Schematic workflow for the co-production of Flor and Isoflor from *P.haitanensis*

Herein, we established a novel four-step integrated chromatographic workflow to simultaneously co-produce high-purity Flor and Isoflor monomers from *P. haitanensis* (Fig. 1). The hepatoprotective activities of the two monomers were assessed in zebrafish and HFD-induced murine MASH models, and their potential mechanisms were further elucidated via multi-omics analyses.

We used high-purity Flor and Isoflor monomers prepared through our scalable co-production workflow from *P. haitanensis* to first perform systematic in vivo biosafety evaluation and lipid-lowering activity screening in zebrafish. To uncover the anti-MASH mechanism of Flor, we applied a comprehensive multi-omics approach integrating network pharmacology, hepatic transcriptomics, gut metagenomics and targeted bile acid metabolomics in HFD-induced murine MASH models. Follow-up validation tests, including in vitro incubation experiments, molecular docking, molecular dynamics (MD) simulations and western blotting (WB), further demonstrated that Flor significantly elevates the relative abundance of intestinal *Parabacteroides goldsteinii* (*P. goldsteinii*). This strain is a critical regulator for the biosynthesis of ursodeoxycholic acid (UDCA), a secondary bile acid. By antagonizing the enterohepatic FXR signaling axis, UDCA sustains the homeostasis of non-12-OH bile acids.

## Materials and methods

### Chemicals and materials

Floridoside standards were obtained from Sigma-Aldrich (St. Louis, MO, USA). Analytical-grade reagents were provided by Sinopharm (Shanghai, China), and HPLC-grade solvents were sourced from Thermo Fisher Scientific (USA). Ultrapure water was prepared with a Milli-Q system (Millipore, Bedford, MA, USA).

### Co-production of Flor and Isoflor

*Pyropia haitanensis* samples were collected from the mid-to-high intertidal zone of Nanji Island, Wenzhou, China (27° 27′ 10″ N, 121° 04′ 58″ E) in November 2024. Following species identification, the specimens were freeze-dried until constant weight, ground into powder, and preserved at − 80 °C for subsequent extraction. Full details of the preparation procedure are provided in Supporting Information S1.1.

Briefly, 1000 g pretreated algal powder was extracted in triplicate with 72.3% ethanol at 60 °C and 200 rpm for 4 h, with a solid–liquid ratio of 1:5 (w/v). All filtrates were pooled and concentrated, then sequentially extracted three times with ethyl acetate to eliminate lipophilic contaminants. The resulting aqueous fraction was harvested and re-concentrated.

The concentrate was successively loaded onto activated Dowex 50W × 8 (H⁺) cation-exchange and Dowex 1 × 8 (Cl⁻) anion-exchange columns, and eluted with ultrapure water. All target fractions were pooled, concentrated and lyophilized to afford the primary crude extract.

Further purification was carried out on a preparative RP-C18 column with methanol–water gradient elution at a flow rate of 15 mL/min and UV detection at 200 nm. Fractions corresponding to Flor and Isoflor (4.75–5.75 min) were collected and concentrated (Table S1 lists the detailed gradient conditions). The two isomers were then separated on a preparative NH₂ column using an acetonitrile–water mixture (80:20, v/v) under isocratic elution at 10 mL/min. Flor (18.5–20.5 min) and Isoflor (23.0–25.0 min) were collected individually, combined and lyophilized to obtain pure white powdered monomers (Table S2 summarizes the NH₂ isocratic elution parameters).

### In vivo* evaluation in zebrafish*

Adult AB-strain zebrafish (3 months of age, body weight: 0.30 ± 0.02 g, male to female ratio = 1:1) were obtained from Shanghai FishBio Co., Ltd. (Shanghai, China). Detailed protocols for zebrafish rearing, as well as the biosafety and lipid-lowering activity assessments of Flor and Isoflor, are available in Supporting Information S2.1.

Briefly, adult zebrafish were reared in a standard recirculating aquaculture system under tightly regulated temperature, photoperiod and water quality. Post-spawning embryos were collected, rinsed and incubated, and abnormal or unfertilized embryos were discarded. MAFLD was induced in 5 days post-fertilization (dpf) larvae via 72 h daily exposure to egg yolk and glucose. The larvae were randomly grouped (30 individuals per group): normal control group, MAFLD model group, positive control group (atorvastatin), and treatment groups administered with serially diluted Flor or Isoflor (2.5, 5, 10 mg/100 mL). Hepatic lipid deposition was detected by Oil Red O (ORO) staining, and the staining intensity was quantified with LAS X software. For in vivo toxicity testing, five larvae per group underwent 72 h continuous locomotor activity tracking to assess neurobehavioral toxicity. Hemodynamic parameters were measured post-treatment to evaluate cardiovascular toxicity.

### In vivo* evaluation in mice*

#### Mice MASH model and experimental design

All animal experiments were approved by the Animal Ethics Committee of Ningbo No. 2 Hospital (Ethics Approval No. GK-2025-XM-0139) and complied with national laboratory animal welfare guidelines. A total of forty male C57BL/6 mice (7 weeks of age, body weight: 20 ± 1.0 g) were purchased from Zhejiang Weitong Lihua Laboratory Animal Co., Ltd., China. Animals were randomly divided into a normal control group (standard diet, n = 8) and an HFD group (n = 32). After 28 weeks of HFD feeding to induce MASH, the HFD mice were re-randomized into four subgroups (n = 8 per group): model group (Mod, HFD alone), low-dose floridoside group (L-Flor, 70 mg/kg), high-dose floridoside group (H-Flor, 200 mg/kg), and positive control group (Res, 3.0 mg/kg resmetirom). Intragastric administration was performed once daily for 8 weeks, and the whole experiment lasted for 36 weeks. Body weight was monitored biweekly during treatment. After a 12 h overnight fast, all mice were euthanized by cervical dislocation. Orbital blood samples were collected for serum analysis, liver tissues for histopathology and molecular detection, and colonic feces for gut microbiota profiling.

#### Biochemical and histopathological analysis

Serum levels of alanine aminotransferase (ALT), aspartate aminotransferase (AST), triacylglycerol (TG) and total cholesterol (TC) were quantified using commercial kits from Nanjing Jiancheng Biological Co., Ltd. (Nanjing, China). Hepatic TG and hydroxyproline (HYP) contents were assayed per standard protocols, with all tests conducted in triplicate.

Liver tissues were fixed in 4% paraformaldehyde for 48 h, followed by dehydration, paraffin embedding and sectioning. Paraffin sections were stained with H&E and Sirius Red, and frozen sections were stained with ORO for the assessment of lipid deposition. For immunohistochemical staining, paraffin sections underwent dewaxing, rehydration, antigen retrieval and blocking with 3% H₂O₂ and serum. Subsequently, the sections were incubated with primary antibody against α-smooth muscle actin (α-SMA), incubated with secondary antibody, and counterstained with hematoxylin. All specimens were visualized using a Nikon Ni-U inverted microscope. Image acquisition and quantitative analysis were performed with HSView software.

### Cell experimental design

HepG2 cells were maintained in Dulbecco’s Modified Eagle Medium (DMEM) containing 10% fetal bovine serum (FBS) and 1% penicillin–streptomycin under standard culture conditions. To investigate the lipid-lowering effect of Flor in vitro, cells were seeded into 6-well plates and assigned to four groups: normal control, model group (250 μM sodium palmitate combined with 500 μM sodium oleate), positive control group (1 μM resmetirom), and Flor-treated group (200 μM Flor alongside the above fatty acids). Following 48 h incubation, ORO staining was performed to visualize intracellular lipid deposition under a light microscope.

### Transcriptomics analysis

Total RNA was extracted from hepatic tissues with TRIzol reagent (Invitrogen, USA). The purity, concentration and integrity of RNA were determined using a NanoDrop 2000 spectrophotometer and an Agilent 2100 Bioanalyzer (Agilent Technologies, USA). Samples with RIN ≥ 7 were used for RNA-seq library construction with the VAHTS Universal V10 RNA-seq Library Prep Kit (Vazyme, China). Transcriptomic sequencing and subsequent bioinformatic analyses were performed by OE Biotech Co., Ltd. (Shanghai, China).

### Metagenomics sequencing and gut microbiota analysis

Total microbial DNA was extracted from mouse fecal samples using the MagPure Soil DNA LQ Kit (Magen, China). DNA integrity and purity were validated with an OD₂₆₀/₂₈₀ ratio ranging from 1.8 to 2.0 and clear electrophoretic bands. Qualified DNA was ultrasonically sheared, and paired-end libraries were prepared with the NEBNext Ultra II DNA Library Prep Kit. Library concentrations were quantified using the Qubit dsDNA Assay Kit (Life Technologies, USA). Shotgun metagenomic sequencing was performed on the Illumina NovaSeq 6000 platform by OE Biotech Co., Ltd. (Shanghai, China).

Raw sequencing reads were filtered to generate clean reads, followed by de novo assembly via MEGAHIT (v1.2.9). Prodigal (v2.6.3) was used to predict open reading frames (ORFs) from the assembled contigs. Sequence alignment against the NCBI NR database was conducted using DIAMOND (v2.1.9) for taxonomic classification. Alpha diversity analyses and data visualization were completed using R software (v4.1.2).

### Targeted bile acid metabolomic analysis

Fifty-three BA reference standards (purity > 95%) were purchased from Shanghai Zzbio Co., Ltd. (Shanghai, China). Cholic acid-d4 was used as the internal standard for quantification. BAs in mouse liver samples were analyzed by ultra-high performance liquid chromatography-tandem mass spectrometry (UHPLC-MS/MS). The analysis was performed on a Waters UHPLC system coupled to an AB Sciex Qtrap 5500 mass spectrometer, with chromatographic separation on a Phenomenex Kinetex C18 column (2.1 mm × 100 mm, 2.6 µm) at 45 °C.

The mobile phase was composed of 0.1% (v/v) formic acid in water (solvent A) and a 1:1:1 (v/v/v) mixture of methanol, acetonitrile and isopropanol containing 0.1% (v/v) formic acid (solvent B). The flow rate was 0.45 mL/min, and the injection volume was 2 µL. The gradient elution program was: 0–0.5 min, 80% A/20% B; 0.5–1.5 min, linear gradient to 62% A/38% B; 1.5–12 min, linear gradient to 50% A/50% B; 12–17.5 min, linear gradient to 5% A/95% B; 17.5–19 min, 5% A/95% B; 19.01–20 min, re-equilibration with 80% A/20% B.

MS detection was conducted in both positive and negative ESI modes, with parameters set as: curtain gas 35 psi, collision-activated dissociation medium, ion spray voltage 5500 V (positive)/4500 V (negative), ion source temperature 450 °C, ion source gas 1 and gas 2 both 55 psi.

### Network pharmacology, molecular docking and molecular dynamics

Potential targets of Flor were retrieved from the SwissTargetPrediction and PharmMapper databases. MASH and bile acid metabolism-related targets were obtained from the GeneCards database, and all target names were standardized via the UniProt database. Intersecting targets among Flor, MASH and bile acid metabolism were identified by Venn diagram analysis. A protein–protein interaction (PPI) network of overlapping targets was constructed using the STRING database and visualized with Cytoscape (v3.9.1). Key targets were further screened by integrating liver transcriptome differential gene expression data, with expression profiles visualized via a heatmap.

Molecular docking simulations were performed using AutoDockTools (version 1.5.7) to characterize the binding interactions between the ligands (Flor and core bile acids) and the selected key targets. The optimal docking poses and intermolecular interaction profiles were visualized with PyMOL (version 2.5). Subsequently, all-atom MD simulations were conducted in GROMACS (version 2023.2) to validate the conformational stability of the ligand–protein complexes, with a 100 ns production MD run performed at a constant temperature of 300 K and pressure of 1 bar.

### Western blot (WB) analysis

Liver tissues were lysed on ice using RIPA lysis buffer (Beyotime Biotechnology, Shanghai, China) supplemented with 1 mM phenylmethylsulfonyl fluoride (PMSF, Beyotime Biotechnology) for 30 min, with vortex mixing every 5 min to ensure complete lysis. The tissue lysates were centrifuged at 14,000×*g* for 15 min at 4 °C, and the supernatants containing total protein were collected. The total protein concentration of each sample was determined using a Bicinchoninic Acid (BCA) Protein Assay Kit (Thermo Fisher Scientific, Waltham, MA, USA).

Protein extracts were adjusted to a uniform concentration of 5 mg/mL, mixed with 5 × SDS-PAGE loading buffer (Beyotime Biotechnology), and denatured by boiling at 100 °C for 10 min. Equal amounts of total protein (30 μg per lane) were separated via 10% sodium dodecyl sulfate–polyacrylamide gel electrophoresis (SDS-PAGE), and then electrotransferred onto polyvinylidene difluoride (PVDF) membranes.

The membranes were blocked with 5% non-fat milk dissolved in Tris-buffered saline with Tween 20 (TBST) for 2 h at room temperature, then incubated overnight at 4 °C with the following primary antibodies: anti-CYP27A1 (A23250, ABclonal, Wuhan, China), anti-farnesoid X receptor (FXR, A24015, ABclonal), anti-CYP7B1 (A17872, ABclonal), anti-CYP7A1 (A22897, ABclonal), and anti-CYP8B1 (DF4762, Affinity Biosciences, Jiangsu, China). Anti-GAPDH antibody (LF211, Epizyme, Shanghai, China) was used as an internal loading control.

After washing 3 times with TBST (5 min each time), the membranes were incubated with horseradish peroxidase (HRP)-conjugated goat anti-mouse secondary antibody (LF101, Epizyme) or HRP-conjugated goat anti-rabbit secondary antibody (LF102, Epizyme) for 1 h at room temperature. Following 3 additional washes with TBST, the protein bands were visualized using an enhanced chemiluminescence (ECL) substrate kit, and the relative gray value of each band was quantified using ImageJ software.

### Culture of *Parabacteroides goldsteinii*

*Parabacteroides goldsteinii* (*P. goldsteinii*, strain ATCC BAA-1180) was obtained from the American Type Culture Collection (ATCC, USA). The bacterium was cultured anaerobically at 37 °C in pre-reduced thioglycolate medium with a gas mixture of 90% N₂, 5% H₂ and 5% CO₂. Cells at the exponential phase were diluted to an OD₆₀₀ of 0.1, then treated with floridoside at concentrations of 2, 5 and 10 mg/mL in 96-well plates (6 replicates per group). Blank and solvent control groups were set up in parallel. Bacterial growth was tracked by OD₆₀₀ readings throughout the 48 h incubation. At 48 h, colony-forming unit (CFU) assays were performed to quantify viable bacteria.

### Cellular thermal shift assay (CETSA) for in situ target engagement validation of UDCA

Cells were seeded into six culture dishes and incubated until reaching 70%–90% confluence. Cells were then treated with 20 μM UDCA or vehicle control at 37 °C for 24 h. All subsequent steps were carried out on ice to avoid protein degradation. After centrifugation (1200 rpm, 3 min, 4 °C), cell pellets were rinsed with ice-cold PBS and resuspended in ice-cold PBS containing 1 × protease inhibitor cocktail at 5 × 10⁷ cells/mL. The cell suspension was divided into aliquots of 100 μL each. Each aliquot was heated from 37 °C to 65 °C at 4 °C increments for 4 min, and then underwent three freeze–thaw cycles (30 s in liquid nitrogen, followed by thawing at room temperature). Cell lysates were centrifuged at 20,000 g for 20 min at 4 °C to pellet denatured aggregates, and the soluble supernatants were harvested. Samples were mixed with 5 × SDS loading buffer and denatured at 95–100 °C for 5–10 min prior to Western blot detection of FXR. Band density was quantified to establish thermal stability curves.

### Statistical analysis

Statistical analyses were performed with IBM SPSS Statistics 26.0 (IBM, USA). Data normality was verified using the Kolmogorov–Smirnov test. One-way ANOVA coupled with Tukey’s post-hoc test was used for normally distributed datasets. The Kruskal–Wallis test with Benjamini–Hochberg FDR correction was adopted for non-normal data. Pearson correlation analysis was used to explore the relationships between gut microbiota, bile acids and MASH-associated metabolic indices. Correlation heatmaps were visualized via the corrplot package in R v4.1.2 and RStudio v2023.06.0. Graphical outputs were generated in GraphPad Prism 9.0. Statistical significance was defined as a two-sided *P* < 0.05 (*P < 0.05, **P < 0.01, ***P < 0.001, ****P < 0.0001).

## Results

### Separation and identification of Flor and Isoflor

UHPLC-Q-Orbitrap-HRMS results verified that lyophilized *P. haitanensis* contains abundant Flor and Isoflor, with respective contents of 6.6 mg/g and 32.1 mg/g dry weight. These values are considerably higher than those documented for other red algal species [18], indicating that this alga is an excellent raw material for isolating the two target isomers.

**Fig.2:**
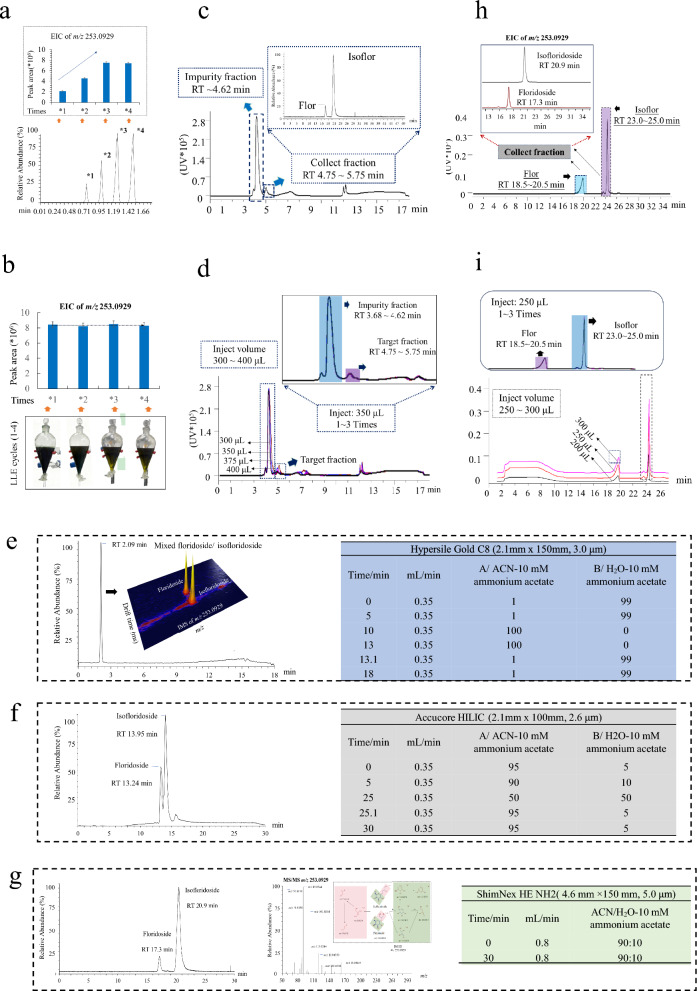
Separation and identification of Flor and Isoflor. **a** Recovery of mixed Flor and Isoflor with extraction cycles 1–4; **b** Effect of liquid–liquid extraction (LLE) cycles (1–4) on compound recovery and lipochrome removal; **c** Chromatographic separation window of target compounds (4.75–5.75 min) from impurities (~ 4.62 min) on a preparative reversed-phase C18 column; **d** Effect of injection volume (300–400 μL) on C18 column chromatographic separation, and reproducibility at 350 μL injection volume (inset); **e**–**g** Comparison of Flor and Isoflor separation using three different columns: **e** Hypersil Gold C8 (2.1 mm × 150 mm, 3.0 μm), **f** Accucore HILIC (2.1 mm × 100 mm, 2.6 μm), **g** ShimNex HE NH₂ (4.6 mm × 150 mm, 5 μm); **h** Chromatographic separation window of Flor (18.5–20.5 min) and Isoflor (23.0–25.0 min) on a preparative normal-phase NH₂ column; **i** Effect of injection volume (200–300 μL) on NH₂ column chromatographic separation, and reproducibility at 250 μL injection volume (inset)

Based on the Flor extraction protocol reported by Gao et al. [5], we systematically optimized extraction cycles, liquid–liquid extraction times, and elution conditions for two ion-exchange resins (detailed optimization procedure see Supporting Information S1.1). Three extraction cycles achieved a 94.3% total recovery of Flor and its isomer (Fig. 2a). Three cycles of equal-volume ethyl acetate liquid–liquid extraction effectively removed pigment impurities without affecting the target compounds (Fig. 2b), which was critical to avoid interference in subsequent chromatographic purification. Under the optimized conditions, a total extraction rate of 3.65% was obtained, higher than the previously reported 3.46% [5], while maintaining the 94.3% recovery. Subsequent ion-exchange resin purification further enriched the target compounds to 49.5% purity in the crude extract (elution conditions see Supporting Information S1.2, Fig. S1).

Reversed-phase C18 chromatography further raised the combined purity of Flor and Isoflor to 94%. These two highly hydrophilic compounds exhibit weak retention on C18 media, so this step mainly served for sample concentration instead of isomeric separation. At the optimal loading volume of 350 μL, the two isomers co-eluted at 4.75–5.75 min. Detailed gradient parameters, loading optimization results and repeatability data are presented in Supporting Information S1.3, Fig. 2c and d.

The final isomeric separation method was initially developed on a ShimNex HE NH₂ analytical column (4.6 × 150 mm, 5 μm). Isocratic elution with acetonitrile/water (80:20, v/v) at 0.8 mL/min enabled the complete baseline separation of Flor and Isoflor (column comparison data in Supporting Information S1.4.1, Fig. 2e–g). To achieve preparative scale-up, a preparative-grade Shim-pack GIST-NH₂ column (250 mm × 20 mm, 5 μm) featuring the same stationary phase was used to maintain stable and reproducible separation performance. With consistent elution parameters, an optimal loading volume of 250 μL, and a flow rate of 10 mL/min, high-purity floridoside (18.5–20.5 min, 99.0% purity) and isofloridoside (23.0–25.0 min, 99.2% purity) were efficiently purified (detailed separation conditions and purity validation data are provided in Supporting Information S1.4.2–S1.4.3 and Fig. 2h–i). This orthogonal separation strategy, integrating C18 reversed-phase enrichment and NH₂ normal-phase purification, effectively overcomes the bottleneck in separating highly polar isomers with highly similar chemical structures.

The chemical identities of purified Flor and Isoflor were confirmed by MS/MS analysis. Both compounds exhibited a deprotonated molecular ion [M–H]⁻ at *m/z* 253.0929 and produced characteristic fragment ions at *m/z* 119.0350 and 89.0244, which were consistent with previously reported data [2]. Their retention times were 17.3 min and 20.9 min, respectively (Fig. 2g). The structures of the two monomers were further validated by ^1^H and ^13^C NMR spectroscopy (NMR data summarized in Table S3). Specifically, Flor was identified as α-D-galactopyranosyl-(1 → 2)-glycerol, while Isoflor was characterized as α-D/L-galactopyranosyl-(1 → 1)-glycerol, consistent with published spectroscopic results. Detailed instrumental parameters are provided in Supporting Information S1.5. Using the established extraction and purification workflow, high-purity Flor (2.07 g/kg dry algal weight, purity ≥ 99.0%) and Isoflor (12.1 g/kg dry algal weight, purity ≥ 99.2%) were successfully acquired.

### In vivo* evaluation using zebrafish and mouse MASH disease models*

**Fig. 3:**
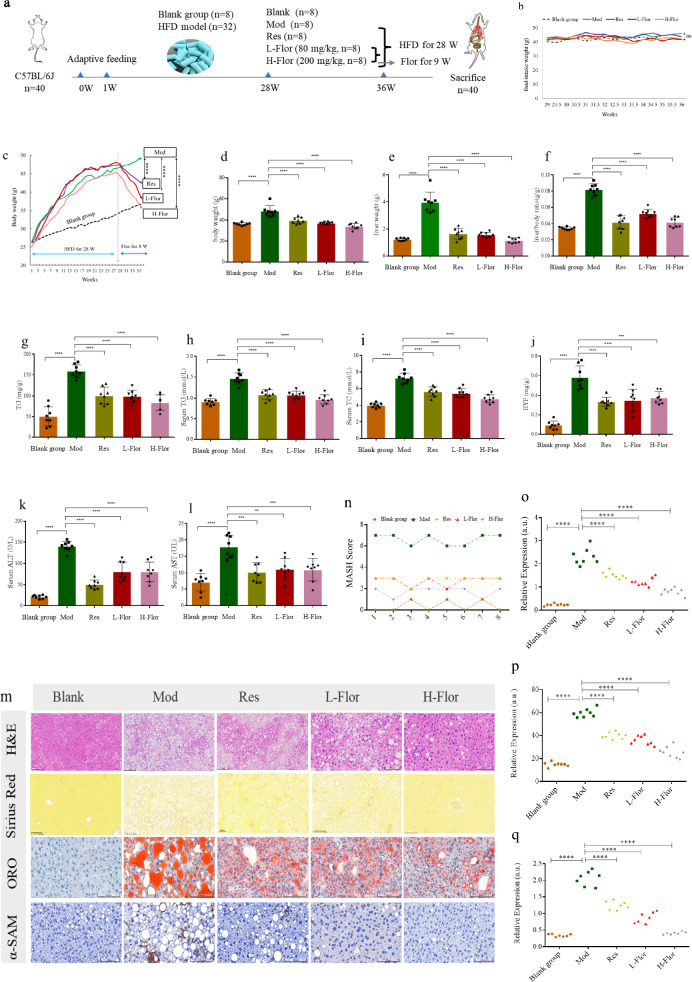
Flor improved liver morphology, liver function, and lipid metabolism in HFD-induced MASH models. **a** Experimental workflow; **b** Food intake; **c** Body weight change curve; **d**–**f** The change trends of body, liver weights and liver-to-body ratio at different group; **g** Hepatic TG levels; **h** Serum TG levels; **i** Serum TC levels; **j** Hepatic HPY levels; **k** Serum ALT levels; **l** Serum AST levels; **m** H&E (Lines 1), Sirius Red (Line 2) and ORO staining (Line 3) (scale bar: 100 μm); **n** MASH score based on H&E staining; **o** Quantification of Sirius Red-positive area; **p** Quantification of ORO-positive area; **q** Quantification of α-SMA-positive area by immunohistochemical staining. All values are expressed as mean ± SD. * indicates statistical difference, *P < 0.05, **P < 0.01, ***P < 0.001, ***P < 0.0001

In the egg yolk-glucose-induced MASH zebrafish model, high-dose Flor and Isoflor exerted concentration-dependent lipid-lowering effects comparable to atorvastatin, with no detectable toxicity (detailed results are provided in Supporting Information S2.2, Fig. S2). Unlike Flor, Isoflor existed as a mixture of chiral isomers rather than a single homogeneous compound. Since the separation and purification of these chiral isomers was not achieved in the present study, Flor was selected for subsequent in-depth mechanistic investigations. To further validate the lipid-lowering efficacy and underlying mechanisms of Flor in mammals, we performed in vivo experiments using an HFD-induced MASH mouse model. The overall experimental timeline is illustrated in Fig. 3a. The entire study lasted 36 weeks, consisting of 28 weeks of HFD-induced model establishment followed by 8 weeks of low- and high-dose Flor intervention. No significant differences in food intake or general physical status were observed among groups throughout the experimental period (Fig. 3b). Flor treatment markedly reversed the MASH-induced increases in body weight, liver weight, and liver-to-body weight ratio (Fig. 3c–f). Serum lipid analysis further demonstrated that Flor significantly reduced the abnormal accumulation of TG and TC, thereby effectively ameliorating dyslipidemia (Fig. 3g–i).

To evaluate liver function, serum ALT and AST levels as well as hepatic HYP content were determined. These biomarkers were markedly upregulated in the model group, indicating severe hepatic injury, whereas Flor treatment significantly reversed these elevations (Fig. 3j–l).

Histopathological examination confirmed severe hepatic steatosis and massive lipid droplet accumulation in mice fed a high-fat diet, as visualized via ORO staining. H&E staining, Sirius Red staining and α-SMA immunohistochemistry further revealed prominent hepatocellular ballooning, disrupted tissue structure and excessive collagen deposition (Fig. 3m). Flor treatment mitigated all these pathological lesions in a dose-dependent fashion (Fig. 3n–q). Overall, our findings demonstrate that Flor exerts a protective effect against HFD-induced MASH.

### *Flor ameliorates MASH in a gut microbiota-dependent manner *via* enrichment of P. goldsteinii*

**Fig. 4:**
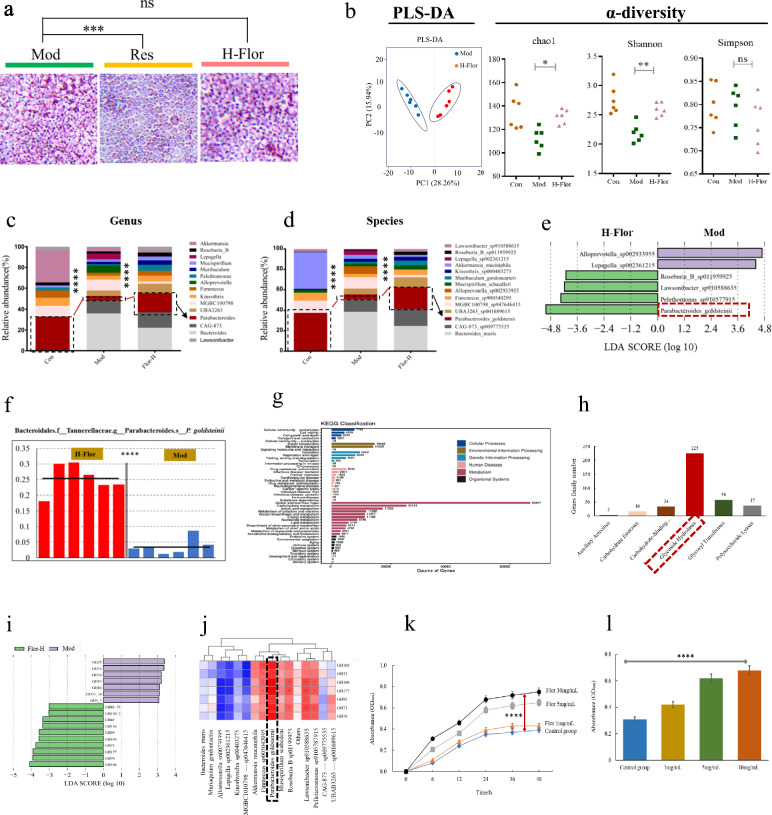
*P. goldsteinii*-encoded glycoside hydrolases mediate the utilization of Flor. **a** Flor did not inhibit OA/PA-induced lipid accumulation in HepG2 cells; **b** PLS-DA of genus-level microbiota and alpha diversity indices of fecal microbiota (n = 6); **c** Relative abundance of bacterial genera among the three groups (*Parabacteroides* indicated by red box); **d** Relative abundance of bacterial species among the three groups (*P. goldsteinii* indicated by red box); **e** Bacterial species enriched in the Flor group vs. model group (LDA > 4); **f** Comparison of *P. goldsteinii* relative abundance between groups; **g** KEGG functional classification of the fecal metagenome; **h** Distribution of GH family genes in the gut microbiome.; **i** LEfSe analysis of CAZy families in the Flor-H group; **j** Pearson correlation between P. goldsteinii abundance and GH family genes; **k** The growth curve of *P. goldsteinii*; **l** The viable counts of *P. goldsteinii*. *P < 0.05, **P < 0.01, ***P < 0.001, ****P < 0.0001

We evaluated the impact of Flor on lipid deposition in HepG2 cells (Fig. 4a). Flor treatment showed negligible effects on lipid accumulation, suggesting that its protective action against MASH is not mediated by direct regulation of hepatocytes. As inter-organ communication, particularly the gut-liver axis, is essential for metabolic homeostasis, we proposed that Flor alleviates hepatic steatosis through modulation of the gut-liver metabolic axis.

To determine how Flor modulates gut microbiota composition, we performed metagenomic sequencing on cecal contents from the control, model and Flor-treated groups. Partial least squares discriminant analysis (PLS-DA) revealed clear and statistically significant clustering of intestinal microbial communities between the model and Flor groups, indicating obvious differences in gut microbiota after Flor intervention (Fig. 4b). For α-diversity analysis, the Simpson index showed no significant differences across the three groups, while the Shannon and Chao indices were elevated upon Flor treatment (Fig. 4b). At the genus level, the abundance of *Parabacteroides* increased markedly after Flor administration (Fig. 4c). At the species level, a bar plot of microbial relative abundance was generated. *Parabacteroides goldsteinii* (a member of the genus *Parabacteroides*) exhibited a striking increase in the Flor-treated groups (Fig. 4d). Linear discriminant analysis effect size (LEfSe) was applied to screen differential species, with the linear discriminant analysis (LDA) score set at a threshold of 3.0. The results demonstrated that *P. goldsteinii* was the most significantly altered species upon Flor intervention (Fig. 4e). The relative abundance of this species in the model and Flor groups is presented in Fig. 4f.

### P. goldsteinii-encoded glycoside hydrolases mediate the utilization of Flor

We performed functional annotation of metagenomic sequences using three well-established databases: CAZy (Carbohydrate-Active enZymes), KEGG (Kyoto Encyclopedia of Genes and Genomes), and eggNOG (Evolutionary genealogy of genes: Non-supervised Orthologous Groups). Functional classification indicated that annotated genes were predominantly enriched in three primary KEGG pathway classes: Metabolism, Genetic Information Processing, and Environmental Information Processing (Fig. 4g). We further identified 225 genes encoding glycoside hydrolase (GH) families in the gut microbial metagenome. As core enzymes, GHs are responsible for the hydrolysis of glycosidic bonds and are closely linked to carbohydrate metabolism (Fig. 4h).

LEfSe was performed to identify differential functional features between groups, which demonstrated that all 10 detected GH families (GH43_33, GH136_2, GH42, GH136, GH95, GH163, GH73, GH177, GH78, and GH106) were significantly overrepresented in the high-dose Flor group (LDA score > 3.0, Fig. 4i). This set of differentially enriched GH families can therefore serve as robust functional biomarkers to distinguish the intervention and control groups. To further elucidate the correlation between key bacterial taxa and these GH-encoding genes, Spearman’s rank correlation analysis was carried out, which revealed a significant positive correlation between the relative abundance of *P. goldsteinii* and the abundance of the aforementioned GH family genes (Fig. 4j).

To ensure robust, accurate CAZyme annotation, we employed a dual-validation pipeline combining HMMER profile search and DIAMOND sequence alignment against the CAZy database, retaining only CAZy families concordantly identified by both algorithms. This workflow yielded 10 high-confidence GH families. All families exhibited extremely low E-values (1.60 × 10⁻^1^⁶^5^ to 2.40 × 10⁻^3^⁶), well below the standard significance threshold (E-value < 1 × 10⁻^5^), eliminating false positives and validating annotation reliability (Summary of GH genes derived from *P. goldsteinii* in the fecal metagenome in Table S4). Integrated functional and taxonomic annotation traced these enriched GH genes to *P. goldsteinii*. Genomic prediction further confirmed that *P. goldsteinii* encodes these specific GHs for Flor recognition and hydrolysis, indicating this bacterium likely utilizes Flor via these genome-encoded enzymes.

To functionally validate the regulatory effect of Flor on *P. goldsteinii*, we assessed its growth-promoting activity on pure cultures of *P. goldsteinii* through 48 h strictly anaerobic in vitro fermentation, with Flor supplemented at final concentrations of 2, 5, and 10 mg/mL. Flor significantly boosted the proliferation of *P. goldsteinii* in a clear dose-dependent manner. Notably, treatment with 10 mg/mL Flor resulted in a 2.14-fold increase in the final optical density at 600 nm (OD₆₀₀) and a 2.19-fold elevation in viable colony-forming unit (CFU) counts relative to the solvent-only negative control (*P* < 0.001) (Fig. 4k, l). These phenotypic findings align perfectly with our prior metagenomic and CAZyme annotation results, collectively demonstrating that Flor acts as a specific growth substrate to drive the proliferation of *P. goldsteinii*.

### P. goldsteinii shows a positive correlation with MASH-related markers and promotes intestinal UDCA biosynthesis

**Fig. 5:**
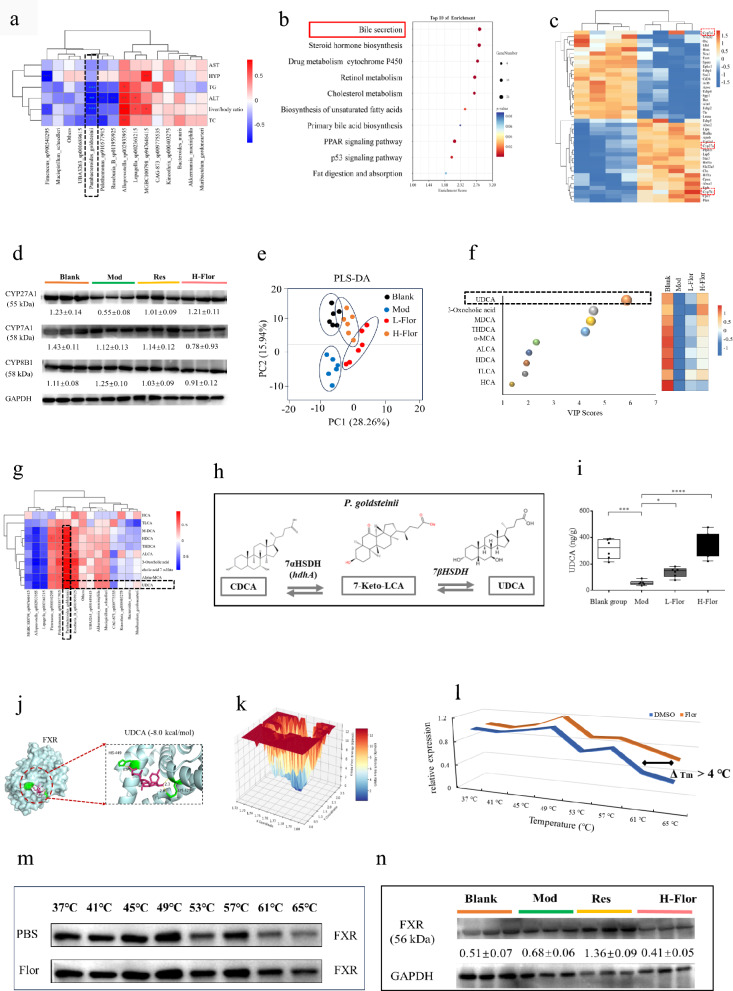
Correlation of *P. goldsteinii* with intestinal UDCA biosynthesis, and molecular docking & dynamics simulations of UDCA with FXR. **a** Pearson correlation between *P. goldsteinii* abundance and MASH-related parameters; **b** RNA-seq functional analysis based on KEGG (Flor group vs. Mod group); **c** Heatmap showing the relative mRNA expression (Flor group vs. Mod group); **d** Protein expression of CYP7A1, CYP27A1, and CYP8B1 in the liver (n = 3); **e** PLS-DA of hepatic BA profiles (Blank, Mod, L-Flor, and H-Flor); **f** VIP scores from PLS-DA of BA profiles (VIP > 4 indicates key metabolites); **g** Pearson correlation between *P. goldsteinii* abundance and BA levels; **h** Intestinal abundance of UDCA; **i** UDCA is synthesized by intestinal microbiota from CDCA and 7-keto-LCA via the *P. goldsteinii*-derived *hdhA* gene; **j** Molecular docking of UDCA with FXR; **k** 3D structural conformation and Gibbs free energy landscape of protein–ligand complexes; **l** CETSA analysis of FXR thermal stability upon UDCA treatment. Western blot showing soluble FXR levels at 37–65 °C; **m** Melting curve of FXR derived from quantitative WB analysis; **n** Intestinal FXR protein expression levels (n = 3). *P < 0.05, **P < 0.01, ***P < 0.001, ****P < 0.0001

We next performed Spearman’s rank correlation analysis to explore the associations between dominant gut microbes and MASH-linked biochemical phenotypes. *P. goldsteinii* was strongly negatively correlated with serum ALT, TG, TC and liver-to-body weight ratio. Conversely, *Alloprevotella* presented positive correlations with these disease-related indices (Fig. 5a). Collectively, these data demonstrate that Flor increases the abundance of *P. goldsteinii*, which in turn markedly mitigates hepatic damage and metabolic dysfunction in MASH models.

Combined multi-omics and target prediction approaches, comprising RNA-seq transcriptomics, disease-associated gene screening and floridoside target identification, indicated that Flor improves MASH phenotypes via modulation of BA homeostasis (see Supporting Information S3.1, Fig. 5b and Fig. S3a–d for full details). Mechanistically, Flor rerouted hepatic BA synthesis away from the canonical CYP7A1-driven classical pathway toward the CYP27A1-dependent alternative pathway. Concordantly, the hepatic alternative BA biosynthesis pathway was significantly upregulated upon Flor treatment (Fig. 5c–d, Fig. S3e).

PLS-DA analysis identified markedly altered BAs (Fig. 5e). UDCA, 3-oxocholic acid, murideoxycholic acid (MDCA) and taurohyodeoxycholic acid (THDCA) were screened as key functional BAs mediating the therapeutic effects of Flor (VIP > 4). These non-12-OH secondary BAs were significantly elevated in the liver following Flor administration (Fig. 5f). Correlation analysis further revealed a significant positive correlation between hepatic levels of these BAs and the abundance of *P. goldsteinii* (Fig. 5g).

In both humans and mice, intestinal microbes produce UDCA from CDCA through the intermediate 7-keto-lithocholic acid (7-keto-LCA). This biotransformation is catalyzed by the protein encoded by the hdhA gene. 7α/β-hydroxysteroid dehydrogenase (7α/β-HSDH), a key enzyme responsible for converting CDCA to 7-keto-LCA and UDCA, has been purified and characterized in *Parabacteroides* species in an earlier study [16]. A recent report further validated the UDCA synthetic ability of *P. goldsteinii* [12] (Fig. 5h). Fecal samples collected from the colon exhibited UDCA variations analogous to those seen in liver tissues (Fig. 5i). Overall, our data demonstrate that *P. goldsteinii* is strongly correlated with intestinal UDCA biosynthesis.

### UDCA directly binds to FXR and negatively regulates FXR signaling activity

Accumulated evidence has indicated that enzymes involved in BA biosynthesis are modulated by the FXR pathway [11]. Molecular docking revealed that UDCA bound stably to FXR, with a binding energy of − 8.0 kcal/mol (Fig. 5j).

We performed MD simulations to verify the molecular docking predictions and evaluate the conformational stability and flexibility of the UDCA-FXR complex. Based on analyses of root mean square deviation (RMSD) (Fig. S3f), root mean square fluctuation (RMSF) (Fig. S3g), hydrogen bond occupancy (Fig. S3h) and Gibbs free energy landscape (Fig. 5k, Fig. S3i), the complex reached a stable conformational state throughout the simulation process (see Supporting Information S3.2 for detailed methods). CETSA results revealed that UDCA markedly elevated the thermal stability of cellular FXR. Relative to the vehicle control, UDCA increased the melting temperature (Tm > 4 °C) of FXR by more than 4 °C and preserved abundant soluble FXR under heat stress, which confirms the direct in situ interaction between UDCA and FXR (Fig. 5l–m). WB analysis further indicated that UDCA inhibited the activation of the FXR signaling pathway (Fig. 5n).

Collectively, these findings support a potential mechanistic pathway whereby Flor-mediated enrichment of *P. goldsteinii* alleviates hepatic injury and metabolic dysfunction in MASH. Specifically, *P. goldsteinii* may facilitate intestinal UDCA biosynthesis, which could in turn drive the synthesis of alternative non-12-OH BAs by suppressing intestinal FXR activity.

### *Flor reversed MASH-induced imbalance between 12-OH and non-12-OH BAs *via* the Parabacteroides goldsteinii–UDCA–FXR axis*

**Fig. 6:**
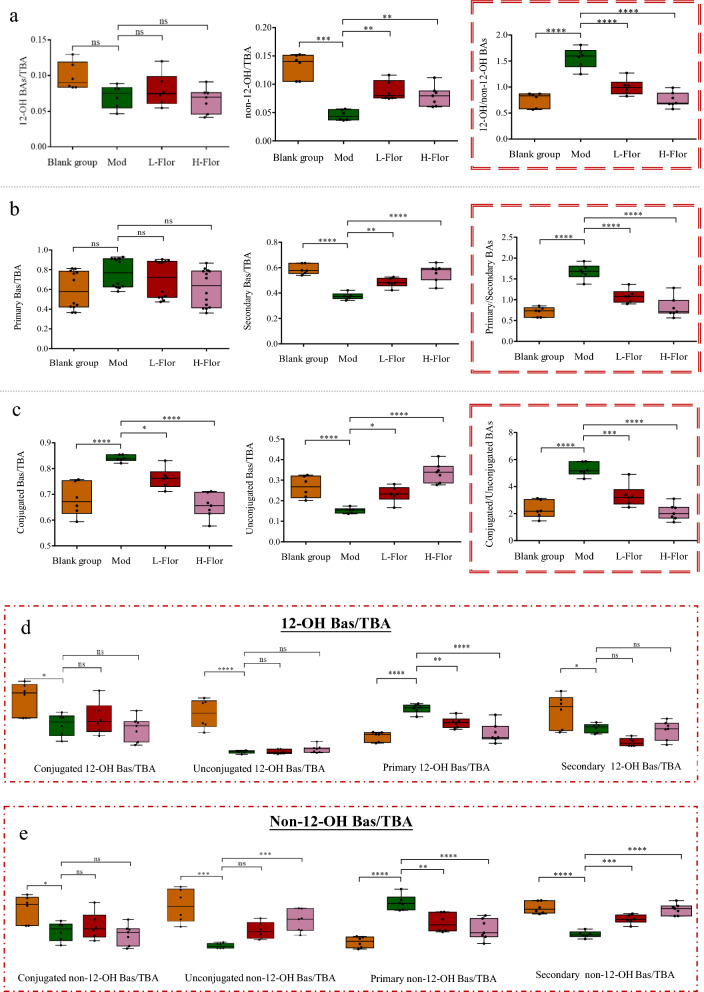
Flor restores the hepatic BA subtype balance disrupted by MASH. **a** Abundances of 12-OH BAs, non-12-OH BAs, and the 12-OH/non-12-OH BA ratio; **b** Abundances of primary BAs, secondary BAs, and the primary/secondary BA ratio; **c** Abundances of conjugated BAs, unconjugated BAs, and the conjugated/unconjugated BA ratio; **d** Compositional profiles of 12-OH BAs; **e** Compositional profiles of non-12-OH BAs. *P < 0.05, **P < 0.01, ***P < 0.001, ****P < 0.0001

To further validate the above hypothesis, hepatic BA profiles were stratified and analyzed based on three classification criteria: 12-OH versus non-12-OH BAs, primary versus secondary BAs, and conjugated versus unconjugated BAs. Ratio analysis revealed that Flor significantly rescued the HFD-induced elevation in the 12-OH/non-12-OH BA ratio (Fig. 6a), indicating a restorative shift toward non-12-OH BA enrichment. Flor also reversed the increased primary/secondary BA and conjugated/unconjugated BA ratios (Fig. 6b, c), thereby narrowing the metabolic differences between the control and Flor groups.

We further analyzed the compositional characteristics of 12-OH and non-12-OH BAs, which are produced through the classical and alternative BA synthesis pathways, respectively, and are tightly linked to MASH pathogenesis. In the Flor group, the proportion of primary non-12-OH BAs relative to total BAs declined, while the proportions of unconjugated and secondary non-12-OH BAs increased. These changes indicated that the overall elevation of non-12-OH BAs was predominantly attributed to the accumulation of their secondary and unconjugated fractions. In contrast, the total and individual 12-OH BA levels remained unaltered in both the model and Flor-treated groups (Fig. 6d, e).

Absolute quantification of distinct BA subclasses yielded consistent results (Fig. S4a–i). Hepatic total bile acid (TBA) levels did not differ significantly across groups. Notably, Flor treatment restored the reduced levels of non-12-OH BAs, secondary BAs, and unconjugated BAs in model mice. Further compositional analysis of 12-OH and non-12-OH BAs confirmed that the elevated total non-12-OH BA content primarily stemmed from the accumulation of unconjugated and secondary BA fractions.

## Discussion

We established a novel four-step integrated process to co-produce high-purity Flor and Isoflor monomers from *P. haitanensis*, with yields of 2.07 g/kg and 12.1 g/kg dry algal weight (purity ≥ 99.0% and ≥ 99.2%, respectively). This work validates *P. haitanensis* as a cost-effective natural source for industrial scale-up, overcomes the technical bottleneck caused by the high structural similarity and water solubility of the two isomers, and breaks the long-standing material barrier for exploring the anti-MASH mechanisms and bacterial targets of Flor.

We first assessed the biosafety and lipid-regulating effects of purified Flor and Isoflor using a zebrafish MASH model. Neither compound exerted obvious toxic effects on zebrafish larvae at concentrations up to 10 mg/100 mL. Given their comparable lipid-lowering potency and the better physicochemical stability of Flor, we selected Flor for all subsequent mechanistic and in vivo investigations. We further verified its long-term protective efficacy in a 36-week HFD-induced murine MASH model, where Flor intervention effectively alleviated diet-induced obesity, systemic dyslipidemia, and hepatic injury.

Our results indicated that Flor did not alleviate MASH through direct regulation of hepatocytes. Accordingly, we shifted our focus to the gut microbiota as a potential mediator. Metagenomic analysis confirmed that Flor treatment substantially increased the relative abundance of intestinal *Parabacteroides* in mice. Accumulating clinical evidence has demonstrated that low *Parabacteroides* levels are closely linked to obesity and MAFLD [11]. Among members of the *Parabacteroides* genus, *P. goldsteinii* displayed the most prominent enrichment upon Flor treatment, and its abundance correlated strongly and negatively with key MASH-related phenotypic indicators.

Further functional metagenomic profiling revealed significant enrichment of multiple GH families in the high-dose Flor group. Spearman’s correlation analysis confirmed a strong positive association between *P. goldsteinii* abundance and these GH genes. Genomic characterization verified that *P. goldsteinii* encodes functional GH enzymes capable of recognizing and hydrolyzing Flor. Consistent with these genomic findings, in vitro fermentation assays demonstrated that Flor specifically promoted the growth of *P. goldsteinii*. Collectively, these results indicate that GH enzymes encoded by *P. goldsteinii* enable this bacterium to utilize Flor, thereby supporting its selective proliferation.

Transcriptomic sequencing further revealed that Flor treatment significantly enriched the BA secretion pathway. *P. goldsteinii* is a well-characterized commensal bacterium that modulates host BA homeostasis [31]. Consistent with this notion, targeted BA profiling of hepatic and colonic contents identified a strong positive correlation between UDCA levels and *P. goldsteinii* abundance in our experimental system. This result is supported by previous findings showing that *P. goldsteinii* directly facilitates UDCA biosynthesis by converting hepatic CDCA to 7-keto-LCA via the enzyme encoded by its hdhA gene [12] (Fig. 5h). Collectively, these observations confirm a robust functional association between *P. goldsteinii* and intestinal UDCA synthesis.

Our in silico analysis, CETSA, and in vitro assays collectively confirm that UDCA functions as a high‑affinity endogenous FXR antagonist, which relieves FXR-mediated transcriptional repression of CYP7B1 and CYP27A1. Consistent with this molecular mechanism, our in vivo study demonstrated that Flor selectively activates the alternative BA synthetic pathway via transcriptional and translational upregulation of these two rate-limiting enzymes, thereby driving a significant elevation in hepatic non-12-OH BA levels. While BA signaling is a promising MASH therapeutic target, no BA-based therapies have been approved clinically due to off-target effects of systemic FXR modulation [13, 15]. Here, we defined a novel gut microbiota-dependent mechanism for the *P. haitanensis*-derived prebiotic Flor against MASH: Flor restores intestinal beneficial *P. goldsteinii* to promote intestinal UDCA biosynthesis, which in turn acts as a tissue-specific FXR antagonist to upregulate the alternative BA synthetic pathway, rebalance the hepatic 12-OH/non-12-OH BA pool, and alleviate MASH-related hepatic steatosis, inflammation and metabolic dysfunction. This work provides robust preclinical validation for Flor as a safe, natural MASH intervention candidate.

## Conclusion

In conclusion, our study resolves two long-standing bottlenecks that have hindered the translational research of Flor: the absence of an industrial process for preparing high-purity Flor monomers, and the undefined molecular mechanism underlying its anti-MASH bioactivity.

We developed a novel four-step integrated co-production process using mass-cultivable *P. haitanensis* as the raw material. This robust workflow yielded high-purity Flor (≥ 99.0% purity, 2.07 g/kg dry algal weight) and its isomer Isoflor (≥ 99.2% purity, 12.1 g/kg dry algal weight), breaking the long-standing material barrier for in-depth functional and mechanistic investigations of these compounds. We first validated the biosafety and lipid-lowering efficacy of both monomers in a zebrafish model, with no detectable toxicity observed even at the maximum tested concentration. We further confirmed that long-term Flor administration significantly ameliorated obesity, systemic lipid dysregulation, hepatic dysfunction, steatosis, and fibrosis in a 36-week HFD-induced murine MASH model, providing robust preclinical evidence for the anti-MASH therapeutic potential of Flor.

Mechanistically, Flor may exert its therapeutic effects via the *P. goldsteinii*-UDCA-FXR enterohepatic axis: Flor treatment enrich the beneficial commensal *P. goldsteinii*, which could in turn promote intestinal UDCA biosynthesis. Microbially derived UDCA may act as an FXR antagonist to derepress the expression of CYP7B1 and CYP27A1—the rate-limiting enzymes of the alternative BA synthetic pathway—thereby likely upregulating hepatic non-12-OH BA levels and reshaping the hepatic BA pool. Notably, distinct from systemic FXR modulators that suffer from severe off-target toxicity, Flor achieves tissue-specific FXR regulation by reshaping the gut microbiota, thereby overcoming the key safety concerns associated with existing experimental MASH therapeutics.

Overall, this work develops an efficient method for the simultaneous purification of high-purity Flor and Isoflor monomers. More importantly, it demonstrates that Flor is a safe and promising natural prebiotic for the prevention and treatment of MASH.

Limitations: the present study only provides a preliminary elucidation of the regulatory mechanism underlying Flor-mediated amelioration of lipid metabolic disorders, and the precise causal links within this regulatory axis remain to be rigorously verified in follow-up in-depth investigations. Accordingly, definitive causal relationships warrant further validation through rigorously designed interventional experiments—including monobacterial colonization assays as well as gene overexpression and knockout animal models—to be conducted in an independent, dedicated round of in vivo studies.

## Supplementary Information

Supplementary material 1.

## Data Availability

No datasets were generated or analysed during the current study.

## Abbreviations

BA: Bile acid
CETSA: Cellular Thermal Shift Assay
CFU: Colony-forming unit
CYP7A1: Cytochrome P450 7A1
CYP7B1: Cytochrome P450 7B1
CYP27A1: Cytochrome P450 27A1
DMEM: Dulbecco’s Modified Eagle Medium
FBS: Fetal bovine serum
Flor: Floridoside
FXR: Farnesoid X receptor
GH: Glycoside hydrolase
HCC: Hepatocellular carcinoma
HFD: High-fat diet
HYP: Hydroxyproline
Isoflor: Isofloridoside
LDA: Linear discriminant analysis
LEfSe: Linear discriminant analysis effect size
MAFLD: Metabolic dysfunction-associated fatty liver disease
MASH: Metabolic dysfunction-associated steatohepatitis
MD: Molecular dynamics
NCBI NR: National Center for Biotechnology Information Non-Redundant protein database
OCA: Obeticholic acid
ORO: Oil Red O
P. goldsteinii: Parabacteroides goldsteinii
PPI: Protein-protein interaction
PVDF: Polyvinylidene difluoride
RMSD: Root mean square deviation
RMSF: Root mean square fluctuation
SDS-PAGE: Sodium dodecyl sulfate-polyacrylamide gel electrophoresis
TC: Total cholesterol
TG: Triacylglycerol
TBST: Tris-buffered saline with Tween 20
UDCA: Ursodeoxycholic acid
UHPLC-MS/MS: Ultra-high performance liquid chromatography-tandem mass spectrometry
WB: Western blot
ALT: Alanine aminotransferase
AST: Aspartate aminotransferase
hdhA: 7α/β-hydroxysteroid dehydrogenase gene
α-SMA: Alpha-smooth muscle actin
CAZy: Carbohydrate-Active enZymes database
KEGG: Kyoto Encyclopedia of Genes and Genomes
eggNOG: Evolutionary genealogy of genes: Non-supervised Orthologous Groups
PLS-DA: Partial least squares discriminant analysis
VIP: Variable importance in projection
TBA: Total bile acid
12-OH BAs: 12α-hydroxylated bile acids
non-12-OH BAs: Non-12α-hydroxylated bile acids
CDCA: Chenodeoxycholic acid
7-keto-LCA: 7-keto-lithocholic acid
MDCA: Murideoxycholic acid
THDCA: Taurohyodeoxycholic acid
ECL: Enhanced chemiluminescence
BCA: Bicinchoninic Acid
PMSF: Phenylmethylsulfonyl fluoride
HRP: Horseradish peroxidase
D₆₀₀: Optical density at 600 nm
FDR: False discovery rate
L-L extraction: Liquid-liquid extraction
NMR: Nuclear magnetic resonance
HRMS: High-resolution mass spectrometry
Q-Orbitrap: Quadrupole Orbitrap mass spectrometer
GAPDH: Glyceraldehyde-3-phosphate dehydrogenase
