# Supplementary material for: Co-production of high-purity floridoside and isofloridoside ameliorates MASH via Parabacteroides goldsteinii-UDCA-FXR enterohepatic axis
Source: Chin Med. 2026 Jul 17;21:197. doi: 10.1186/s13020-026-01450-9 (PMC13377823; doi:10.1186/s13020-026-01450-9)
Supplement: Supplementary file 1 — Supplementary material 1. [file 13020_2026_1450_MOESM1_ESM.docx]

**Supporting Information**

*S1. Detailed Procedure for Co-production of Flor and Isoflor*

*S1.1. Four-Step Chromatographic Co-production of High-Purity Flor and Isoflor Monomers from P. haitanensis*

***Extraction of Crude Extract***

For extraction, 1000 g of pretreated algal powder was extracted three times with 72.3% ethanol (v/v) at a solid-to-liquid ratio of 1:5 (w/v). Each extraction was performed in an orbital shaker at 60 °C, 200 rpm for 4 h. After each cycle, the mixture was filtered through nine layers of sterile gauze, and the filtrates were combined. The combined extract was concentrated under reduced pressure using a rotary evaporator (IKA RV 10, IKA-Werke GmbH & Co. KG, Staufen, Germany) at 50 °C to remove ethanol. To remove lipophilic impurities, the concentrated aqueous extract was mixed with an equal volume of ethyl acetate (1:1, v/v) for liquid-liquid extraction, repeated three times. The upper ethyl acetate phase was discarded, and the lower aqueous phase was collected and concentrated to a final volume of ≤ 100 mL.

***Ion-Exchange Chromatography Purification***

Ion-exchange resins (Dowex 50W×8, H⁺ form; Dowex 1×8, Cl⁻ form, Thermo Fisher Scientific, USA) were activated per the manufacturer’s instructions, and packed into glass columns (30 × 300 mm) to a settled bed volume of 200 mL for each resin. The concentrated aqueous phase from liquid-liquid extraction was diluted to 100 mL with degassed ultrapure water, and filtered through a 0.45 μm hydrophilic polyethersulfone (PES) membrane (Jinteng, Tianjin, China).

The pretreated sample was first loaded onto the activated cation-exchange column, eluted with ultrapure water at ≤ 1 drop/s, with 27 fractions (15 mL each) collected. Fractions 2–15 were pooled based on DART-MS analysis and color change, then concentrated to 100 mL. The eluate was subsequently loaded onto the activated anion-exchange column and eluted under the same conditions. Target fractions (2–14, ~210 mL) were confirmed by DART-MS, pooled, concentrated by rotary evaporation, and lyophilized to obtain the primary crude extract, which was stored at –80 °C until subsequent experiments.

***Enrichment of mixed Flor and Isoflor***

Preparative chromatographic purification was performed on a Shimadzu Essentia LC-16 system (Shimadzu Corporation, Kyoto, Japan) equipped with a diode array detector. The lyophilized crude extract was dissolved in ultrapure water to a final concentration of 100 mg/mL, and filtered through a 0.45 μm PES membrane.

Target Flor and Isoflor were enriched on a preparative reversed-phase C18 column (250 × 20 mm, 10 μm) (Shimadzu Corporation, Kyoto, Japan). Gradient elution was carried out with a methanol (A)-ultrapure water (B) mobile phase system at a constant flow rate of 15 mL/min (Table S1). The injection volume was 350 μL, with detection set at 200 nm. Elution fractions at 4.75–5.75 min containing the target isomer mixture were collected from repeated runs, pooled, and concentrated under reduced pressure at 50 °C for subsequent monomer separation.

**Table S1.** The gradient elution procedure of C18 column

| Time/min | Flow rate (mL/min) | H_2_O/MEOH (%) | Chromatogram |
| --- | --- | --- | --- |
| 0 | 15 | 95/5 | 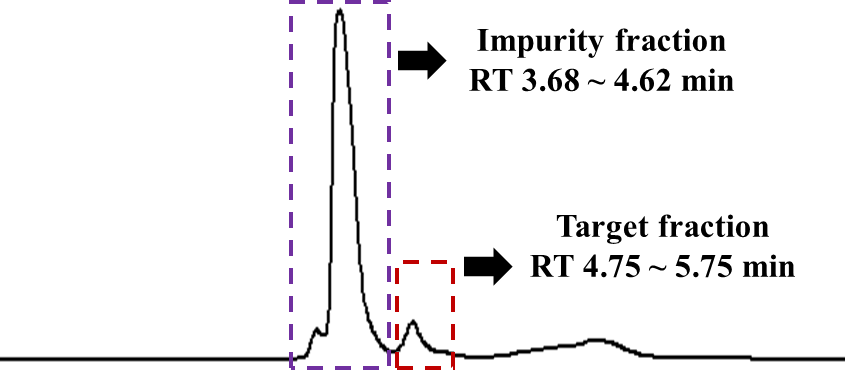 |
| 4.75 | 15 | 95/5 |  |
| 5.75 | 15 | 95/5 |  |
| 6 | 15 | 95/5 |  |
| 8 | 15 | 0/100 |  |
| 12 | 15 | 0/100 |  |
| 12.1 | 15 | 95/5 |  |
| 18 | 15 | 95/5 |  |

***Separation of Flor and Isoflor***

Separation of the two isomers were performed on a Shimadzu Shim-pack GIST-NH2 preparative column (250 × 20 mm, 5 μm) (Shimadzu, Kyoto, Japan). Isocratic elution was adopted with a mobile phase of HPLC-grade acetonitrile and ultrapure water (80:20, v/v) at a constant flow rate of 10 mL/min (Table S2). The injection volume was 250 μL, and the detection wavelength was fixed at 200 nm. The elution fractions corresponding to Flor (retention time: 18.5–20.5 min) and Isoflor (retention time: 23.0–25.0 min) were collected separately, pooled from repeated runs, and lyophilized to obtain white powdered monomeric compounds.

**Table S2.** The isocratic elution procedure of NH_2_ column

| Time/min | Flow rate (mL/min) | H_2_O/ACN (%) | Chromatogram |
| --- | --- | --- | --- |
| 0 | 10 | 20/80 | 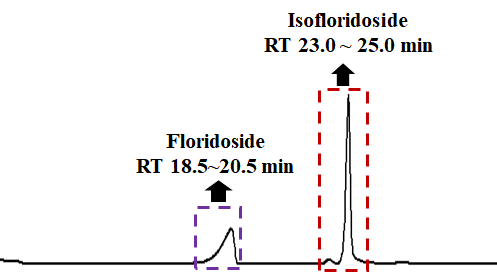 |
| 18.5 | 10 | 20/80 |  |
| 20.5 | 10 | 20/80 |  |
|  | 10 | 20/80 |  |
| 25 | 10 | 20/80 |  |
| 35 | 10 | 20/80 |  |

*S1.2. Optimization of Extraction Prameters*

**Extraction cycle evaluation:** The 1.0 g of lyophilized *P. haitanensis* was extracted with 5 mL of 72.3% ethanol (1:5 solid-to-liquid ratio) at 60°C with agitation (4 h/cycle), followed by centrifugation (3,260 ×g, 5 min). This process was repeated for 1-4 cycles to assess the impact of extraction frequency on target compound recovery. Subsequently, the supernatants were pooled and diluted to the desired final volume with deionized water. Target analytes (floridoside and isofloridoside) were analyzed in real time using matrix-effect-free DART-MS. Quantification was performed by integrating the peak area of their co-eluting characteristic ion (*m/z* 253.0925 [M-H]^-^). All experiments were performed independently in triplicate.

**Liquid-liquid extraction (LLE) optimization**: The 100 mL of concentrated extract was partitioned with an equal volume of ethyl acetate in a separatory funnel. The mixture was shaken for 5 min and allowed to settle for 10 min. The aqueous (upper) and organic phases (lower) were obtained. The extraction process was repeated for 1-4 cycles to systematically evaluate the effects of LLE frequency on both target compound recovery efficiency and lipochrome removal rates. Pigment removal was monitored by visual observation of color change from dark green to light green. The resulting aqueous phases were adjusted to a standardized concentration prior to DART-MS analysis. All experiments were performed independently in triplicate.

the recovery rate

**Calculating extraction rate and recovery rate:** Mixed Flor and Isoflor in the extract were quantified by DART-MS using a 0.5 mg/L floridoside standard after optimal dilution. The extraction rate (EX) of the target analytes in lyophilized *P. haitanensis* biomass (1000 g, W_feed_) were calculated using Eq. (1).

EX (%) = (C_EX_ × V_EX_) × A/ (W_feed_ × 1000) × 100 (1)

where C_EX_ represents the measured concentration of the floridosides in the extract solution (0.76 mg/L), and V_EX_ represents the total volume of the solution (4.0 L), A represents the dilution factor (1.2 × 10^4^). Furthermore, the recovery rate of the floridosides (RE) was calculated using Eq. (2).

RE (%) = (M_EX_ / M_0_) × 100 (2)

where M_EX_ represents the mass of mixed Flor and its isomer in the extract solution (36.5 g/kg), and M_0_ denotes the combined mass of Flor and its isomer in lyophilized *P. haitanensis* (38.7 g/kg, sum of 6.6 and 32.1 mg/g). All measurements were performed in triplicate.

*S1.2. Ion-Exchange Resin Purification*

We employed a dual ion-exchange resin system for comprehensive purification. Key optimized parameters included a 500 mL elution volume (tubes 1-27) for complete target recovery, and wash volumes (0, 100, 200 mL) to minimize residual impurities without column contamination.

The concentrated aqueous phase (100 mL) was loaded onto an activated cation-exchange column. Elution was performed with ultrapure water at ≤1 drop/s, collecting 27 fractions (15 mL each). Fraction 2-15 were pooled based on DART-MS analysis and color change (Fig. S1a, c). Subsequently, the concentrated eluate (tubes 2-15, 100 mL) was similarly processed through an activated anion-exchange column, eluted with ultrapure water (≤1 drop/s) and analyzed by DART-MS (Fig. S1b, d). UV-Vis analysis confirmed negligible impurities in elution tubes 26–27 (Fig. S1e, f), demonstrating that a separate wash step was unnecessary.

Finally, the pooled eluate (tubes 2-14, ~210 mL) was concentrated by rotary evaporation and lyophilized to obtain the primary crude extract, which was stored at -80°C for subsequent purity analysis and further liquid-phase purification.

The purity of both Flor and its isomer (X_flor_) in the process was quantified as a percentage of the lyophilized crude extract biomass, as shown in Eq. (3).

X_flor_ (%) = ( C_flor_ × V_flor_ ) / W_crude_ × 100 (3)

where C_flor_ represents the measured concentration of the Flor and its isomer in the crude extract solution (0.49 mg/L), and V_flor_ represents the volume of the solution (1.0 L), W_crude_ represents the quality of lyophilized crude extract biomass (1.0 mg). All experiments were conducted in three independent replicates.


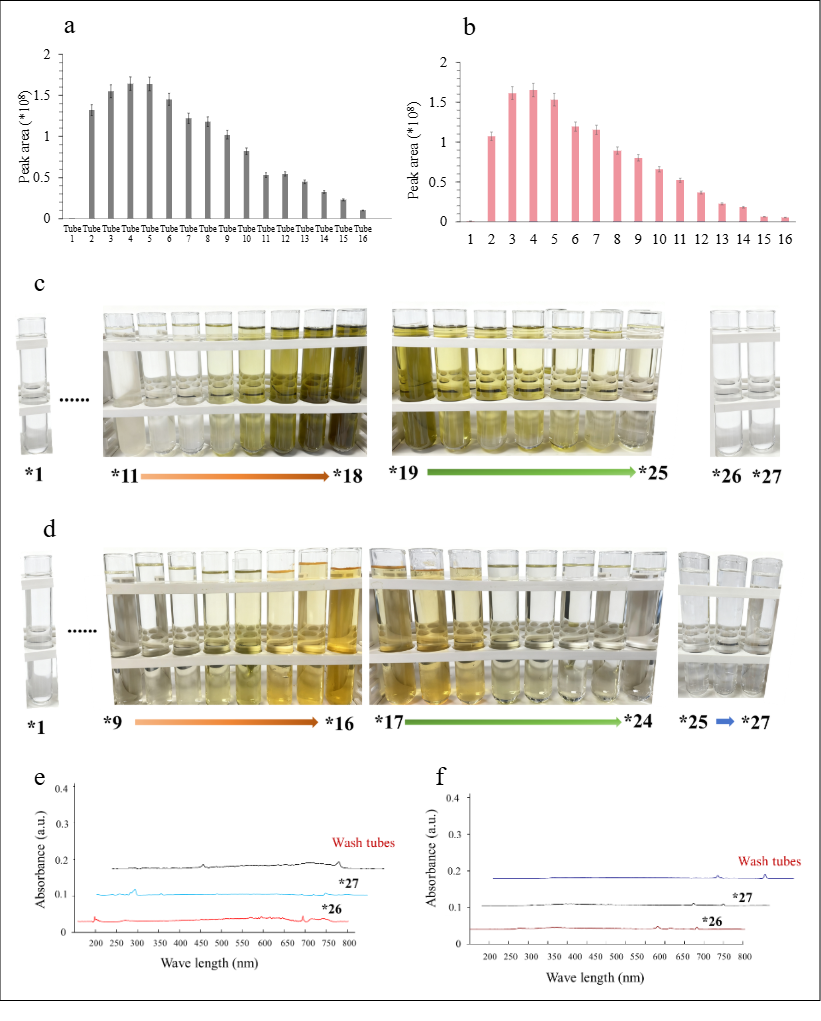


**Fig. S1.** Optimization of Flor and isoflor elution using anion/cation-exchange resins. (a–b) Content distribution after cation (a) and anion (b) exchange purification (n=3); (c–d) color changes during cation (c) and anion (d) exchange purification; (e–f) UV spectra of tubes 26–27 and wash tubes after anion (e) and cation (f) exchange purification.

*S1.3. C18 column Purification and Optimization*

For gradient optimization, the optimal flow rate of 15 mL/min was determined through volumetric scaling calculations derived from the C18 preparative column parameters (250 mm × 20 mm, 10 μm). Employing a methanol (A)/water (B) binary solvent system, the elution gradient was systematically optimized with particular emphasis on extending the high-aqueous phase duration, thereby enhancing resolution between the target compounds and strongly polar impurities. A precisely optimized linear gradient (2% → 5% mobile phase A over 0–6 min) to resolve polar impurities, then changed to 100% methanol over 2 min (6-8 min), coupled with a 4 min isocratic hold, efficiently removed retained nonpolar species from the stationary phase while facilitating the elution of residual components. The system was rapidly re-equilibrated (100% → 2% mobile phase A in 0.1 min) and stabilized for 6 min, ensuring consistent baseline pressure recovery and enabling continuous preparative-scale operation.

For optimization of loading capacity, sample solutions were prepared at 100 mg/mL in water. Injection volumes of 300, 350, 375 and 400 μL were tested. Separation was monitored at 200 nm. Baseline resolution (Rs > 1.5) was maintained up to 350 uL, beyond which peak broadening compromised separation.

For collection window, based on UV detection, fraction eluting between 4.75-5.75 min were collected as the mixed floridosides. Early-eluting fractions (3.68-4.62 min) contained polar impurities and were discarded.

For purity evaluation of the Flor and its isomer mixture, one milligram of the lyophilized purified product was accurately weighed and dissolved in 1.0 L of deionized water. The content of Flor and its isomer was determined by DART-MS. All measurements were performed in triplicate. Final purity (Y_flors_) analysis showed 94.0% combined Flor and its isomer in the lyophilized product (W_purify-flors_), achieving 89.9% impurity depletion (X_impurity_), as shown in Eq. (4) and (Eq.5)

Y_flors_ (%) = ( C_flors_ × V_flors_ ) / W_purify-flors_ × 100 (4)

where C_flors_ represents the measured concentration of the Flor and its isomer in the solution (0.94 mg/L), and V_flors_ represents the volume of the solution (1.0 L), W_purify-flors_ represents the quality of lyophilized purified product biomass (1.0 mg).

X_impurity_ (%) = (1-X_flors_)-(1-Y_flors)_ / (1-X_flors_) × 100 (5)

where X_flors_ (49.5%) represents the purity of combined Flor and its isomer in the lyophilized crude extract, and Y_flors_ (94.0%) represents the purity of those in the lyophilized purified product.

*S1.4. Separation of monomeric floridoside and isofloridoside*

*S1.4.1. Selection of the separation column*

Given that Flor and Isoflor are isomeric compounds that cannot be differentiated by mass spectrometry, chromatographic separation was necessary for their analysis. Thus, three different chromatographic columns with different specifications, namely Hypersil Gold C8 (2.1 mm × 150 mm, 3.0 μm), Accucore HILIC（2.1mm x 100mm, 2.6 μm）and Shimadzu ShimNex HE NH_2_ (4.6 × 150 mm, 5 μm), were compared, and, most importantly, the ability to resolve floridoside and isofloridoside was evaluated.

The C8 column had weak retention of the target compounds, and the effective separation of target compounds could not be achieved by optimizing the composition and gradients of the mobile phase, however, ion mobility imaging enables the clear distinction of floridoside and its isomer. While the HILIC column demonstrated strong retention of hydrophilic compounds, it failed to achieve complete baseline separation of floridoside and its isomer, accompanied by a significant reduction in target compound responses. Optimal separation and peak shape were achieved using a ShimNex HE NH_2_ column (4.6 × 150 mm, 5 μm) with an isocratic normal-phase mobile phase of acetonitrile/water (8:2, v/v) at a flow rate of 0.8 mL/min.

*S1.4.2. Optimization of separation conditions*

For isocratic conditions, the Shim-pack GIST-NH_2_ column was equilibrated with acetonitrile/water (8:2, v/v) at 10 mL/min. Sample (100 mg/mL in water) was injected at 250 μL. Detection at 200 nm showed Flor at 18.5-20.5 min and Isoflor at 23.0-25.0 min.

For loading capacity, injection volumes of 200, 250 and 300 μL were tested. Baseline separation (Rs > 1.5) between Flor and Isoflor was maintained up to the maximum capacity of 250 μL, which was selected as the optimal loading volume.

*S1.4.3. Purity evaluation of the monomeric Flor and Isoflor*

One milligram each of the two lyophilized products (W_flor_ and W_iso-flor_) was accurately weighed and dissolved in 1.0 L of deionized water. The contents of Flor and Isoflor were quantified using UHPLC-Q-Orbitrap-HRMS, showing retention times of 17.3 and 20.9 min, respectively. All analyses were performed in triplicate. The final purity reached 99.0% for Flor (Y_flor_) and 99.2% for Isoflor (Y_iso-flor_), corresponding to 83.3% and 86.7% impurity depletion (X_impurity_), as calculated by Eq. (6) and Eq. (7).

Y_flor/iso-flor_ (%) = ( C_flor/iso-flor_ × V_flor/iso-flor_ ) / W_purify-flor/iso-flor_ × 100 (6)

where C_flor/iso-flor_ represents the measured concentration of the Flor and Isoflor in the solution (0.990 mg/L and 0.992 mg/L), and V_flor/iso-flor_ represents the volume of the solution (1.0 L), W_purify-flor/iso-flor_ represents the quality of lyophilized biomass (1.0 mg).

X_impurity_ (%) = (1-X_flors_)-(1-Y_flor/iso-flor)_ / (1-X_flors_) × 100 (7)

where X_flors_ (94.0%) represents the combined purity of Flor and Isoflor, and Y_flor/iso-flor_ represent the individual purities of Flor and Isoflor, respectively.

*S1.5. Instrumental methods*

**DART-MS analysis:** For rapid quantification of Flor and Isoflor during process optimization, 10 uL of sample was placed on the DART-MS transmission module. Ionization was performed in negative mode with helium gas at 350℃. The ion at *m/z* 253.0929 was integrated for quantification using a 0.5 mg/L Flor standard curve.

**UHPLC-Q-Orbitrap-HRMS:** UHPLC separation was performed on a Thermo Fisher Scientific UltiMate 3000 UHPLC system (Waltham, MA, USA). The UHPLC conditions were as follows: Shimadzu ShimNex HE NH^2^ column (4.6 × 150 mm, 5 μm) was used with isocratic elution (acetonitrile/water, 8:2, v/v) at 0.8 mL/min.

MS analysis was performed on a Q-Exactive Orbitrap mass spectrometer (Thermo Fisher Scientific, USA) equipped with a heated electrospray ionization (HESI) source. The mass spectrometer was operated in ESI⁻ mode with a scan range of *m/z* 50–300, a full scan MS resolution of 120,000, and a data-dependent MS² (dd-MS²) resolution of 35,000. The automatic gain control (AGC) was set to 5.0 × 10⁵ for MS and 2.0 × 10⁵ for dd-MS². The capillary and spray temperatures were 300 °C and 350 °C, respectively. Sheath and auxiliary gas (N₂) pressures were 35 arb and 10 arb, respectively. Normalized collision energy (NCE) was set to 25%, 30%, and 40%.

**Qualitative and quantification:** Qualitative analysis was performed using ExactFinder™ software (Thermo Scientific) based on precursor ion *m/z* (mass error ≤ 5 × 10⁻⁶), retention time, isotopic distribution, and MS/MS spectra, ensuring accurate identification and minimizing false positives.

For quantitation, a calibration curve was constructed using Flor standard solutions (0.02–0.5 mg/L), with concentration as the abscissa and instrument response as the ordinate. The structurally similar analog Isoflor was semi-quantified using the same calibration curve.

**MRM analysis:** Structural elucidation was performed using NMR spectroscopy (Bruker Avance III, 600 MHz, Germany). The compound was dissolved in DMSO‑d₆ or CDCl₃ (10-20 mg/mL), transferred to a 5 mm NMR tube, and analyzed at 25 ± 1 °C. ¹H and ¹³C NMR spectra were acquired at 600 MHz and 150 MHz, respectively, with 64 scans (¹H) and 128 scans (¹³C), and a relaxation delay of 2 s. TMS (δ 0.00 ppm) was used as the internal standard (Srivilai et al., 2014). Data were processed using MestReNova (version 14.0).

**Table S3.** ^1^H and ^13^C NMR data for purified floridoside and isofloridoside

| Position | Floridoside | | Isofloridoside | |
| --- | --- | --- | --- | --- |
|  | ^13^C  (150 MHz) | ^1^H (multiplicity,  *J* = Hz, 600 MHz) | ^13^C  (150 MHz) | ^1^H (multiplicity,  *J* = Hz, 600 MHz) |
| 1 | 99.4, CH | 4.81 (brs, 1H) | 99.3, CH | 4.63 (m, 1H) |
| 2 | 71.4, CH | 3.76 (t, 6.4 Hz, 1H) | 68.6, CH | 3.64 (m, 1H) |
| 3 | 69.8, CH | 3.53 (m, 1H) | 69.3, CH | 3.67 (m, 1H) |
| 4 | 68.8, CH | 3.52 (m, 1H) | 68.8, CH | 3.75 (m, 1H) |
| 5 | 68.9, CH | 3.68 (brs, 1H) | 69.7, CH | 3.71 (m, 1H) |
| 6 | 61.1, CH_2_ | 3.41 (d, 6.7 Hz, 2H) | 63.1, CH_2_ | 3.60 (m, 2H) |
| 1' | 80.6, CH | 3.47 (m, 1H) | 70.3, CH | 3.52 (m, 2H) |
| 2' | 60.6,60.9 CH_2_ | 3.43 (m, 1H), 3.49 (m, 1H) | 71.2, CH | 3.50 (m, 1H) |
| 3' | 60.6,60.9,CH_2_ | 3.43 (m, 1H), 3.49 (m, 1H) | 60.5, CH | 3.56 (m, 2H) |

^*a^Spectra were recorded at 600 MHz for 1H NMR and 150 MHz for ^13^C NMR. The purified floridoside and isofloridoside were dissolved in DMSO-d6, respectively.

*S2. In Vivo Evaluation in Zebrafish*

*S2.1. Zebrafish Rearing and Lipid-Lowering Efficacy Assessment*

***Zebrafish Maintenance and Embryo Collection***

Adult zebrafish (AB line, 3 months old, 0.30 ± 0.02 g, 1:1 male/female) were obtained from Shanghai FishBio Co., Ltd. (Shanghai, China). Fish were housed in a recirculating aquaculture system (Huante Biotech, Hangzhou, China) at 26 ± 2 °C under a 14 h/10 h light/dark cycle, with water quality maintained at pH 7.2 ± 0.5, dissolved oxygen 6.6 ± 0.3 mg/L, conductivity 0.256 ± 0.005 mS/cm, and ammonia nitrogen < 0.02 mg/L. Zebrafish were fed twice daily with freshly hatched brine shrimp and fasted for 12 h prior to spawning. Embryos collected within 1 h post-spawning were rinsed three times with ISO standard embryo medium and incubated at 28.5 °C under a 14 h/10 h light/dark cycle. Unfertilized or malformed embryos were discarded under a stereomicroscope.

***Lipid-Lowering Activity Assessment in Zebrafish***

MAFLD was induced in 5 days post-fertilization (dpf) zebrafish larvae via daily exposure to 0.15 g/100 mL egg yolk (morning) and 0.30 g/100 mL glucose (afternoon) for 72 h. Concurrently, larvae were treated with gradient concentrations of Flor or Isoflor (2.5, 5, 10 mg/100 mL) or positive control atorvastatin (1.16 mg/100 mL), with 30 larvae per group. Untreated normal larvae and model-only larvae were set as the Control group and model group, respectively. Hepatic lipid accumulation was visualized by Oil Red O (ORO) staining, and the corresponding staining intensity was quantified as optical density using LAS X software.

***Toxicity and Biosafety Assessment in Zebrafish***

For *in vivo* toxicity assessment, five normally developed zebrafish larvae (5 days post-fertilization, dpf) were randomly selected from each group at the start of MAFLD induction and drug treatment. Locomotor activity was continuously monitored for 72 h at 28.5 °C using a fully automated video-tracking system (ViewPoint Life Sciences, Lyon, France). Movement trajectories and average swimming speeds were recorded for subsequent analysis. Immediately after the 72 h intervention (8 dpf), hemodynamic parameters including blood flow and blood linear velocity were measured under a stereomicroscope (Leica M205 FA, Leica Microsystems, Wetzlar, Germany) to evaluate cardiovascular toxicity.

*S2.2. Lipid-Lowering Efficacy and Biosafety Evaluation of Flor and Isoflor in Zebrafish*

Zebrafish have emerged as an important model for studying lipid metabolism disorders (Guo et al., 2020; Sapp et al., 2014). Using an egg yolk and glucose-induced MASH model, we evaluated the lipid-lowering effects of Flor and Isoflor (Fig. S2a). Both compounds significantly reduced lipid deposition in a concentration-dependent manner (Fig. S2b-d). At a high concentration (10 mg/100 mL), their efficacy was comparable to that of Ator (1.16 mg/100 mL). Notably, no toxicity was observed at concentrations up to 10 mg/100 mL. All treated groups exhibited 100% survival and no morphological abnormalities. Further assessment using ZebraLab software revealed no significant differences in locomotor trajectories, activity levels (Fig. S2e, f), blood flow (Fig. S2g), or linear velocity (Fig. S2h) between treated and Control groups after 72 h of exposure. Our results revealed that both compounds exhibited excellent in vivo biosafety and comparable lipid-lowering efficacy, with Flor showing a more prominent structure stability, which prompted us to select Flor for subsequent in-depth mechanistic dissection.

**Fig. S2.** Evaluation of the safety and lipid-lowering activity of Flor and Isoflor in zebrafish larvae. (a) Schematic of the experimental workflow; (b) Oil Red O staining in zebrafish larvae treated with Flor; Quantitative analysis of lipid deposition in response to Isoflor (c) and Flor (d) (n=30); Locomotor trajectories and activity levels in response to Isoflor (e) and Flor (f) (n=5); Blood flow (g) and linear velocity (h) in response to Isoflor and Flor (n=5).

*S3. Network Pharmacology and Molecular Dynamics Analysis*

*S3.1. The alternative BA synthesis pathway was markedly activated in Flor-treated groups*

To investigate the global gene expression profiles and identify potential molecular targets underlying the therapeutic effects of Flor, we performed RNA-seq analysis on liver tissues from the blank, Mod, L-Flor, and H-Flor groups. KEGG pathway analysis revealed significant enrichment of the BA secretion pathway. A total of 1,550 MASH-associated targets were collected from public databases, including NCBI, GeneCards, OMIM, and DisGeNET (Fig. S3a). Using SwissTarget, SEA, and PharmMapper, we identified 392 potential target proteins of Flor (Fig. S3b). Integrative analysis of MASH-associated genes (DateBase), Flor-specific differential genes (RNA-seq), and predicted Flor targets (DrugTarget) yielded 20 overlapping candidates as potential targets of Flor in MASH treatment (Fig. S3c). These common targets were imported into the STRING database to construct a protein-protein interaction (PPI) network , in which three genes-cyp27a1, cyp7a1, and cyp7b1-exhibited the highest degree (Fig. S3d). The heatmap of gene abundance showed that Cyp27a1 and Cyp7b1 were markedly upregulated, whereas Cyp7a1 was downregulated, suggesting that Flor intervention shifts BA biosynthesis from the classic to the alternative pathway (Fig. S3e). These findings were validated by WB analysis, confirming the downregulation of CYP7A1 and upregulation of CYP27A1. Collectively, these results demonstrate that CYP27A1-driven alternative BA biosynthesis plays a crucial role in alleviating MASH and represents a key mechanism underlying the therapeutic effects of Flor.

*S3.2. Molecular dynamics simulation of the UDCA-FXR complex*

Root mean square deviation (RMSD) trajectories were analyzed to evaluate the dynamic stability of molecular dynamics (MD) simulations. Generally, lower RMSD values reflect higher complex stability. The blue curve denotes the protein and the red curve denotes the ligand. RMSD values of both components remained within 0.1–0.4 nm, and the ligand maintained a low RMSD of ~0.1 nm, demonstrating tight binding within the complex. These results confirm the stable binding interaction between UDCA and the target protein FXR (Fig. S3f). Root mean square flexibility (RMSF) analysis showed that residue fluctuations in the protein’s core structural domains were all kept below 0.15 nm. Only the N-terminal end and the 330–370 loop region presented mild flexible fluctuations in line with the protein’s native properties, indicating that the protein had a highly stable overall conformation and well-maintained folded state during the simulation (Fig. S3f). Classical hydrogen bonds (blue) were stably maintained at 2–4 for most of the simulation, while polar atom pairs within 0.35 nm (red) fluctuated between 2–10. The persistent hydrogen bond interactions confirm stable ligand-protein binding, consistent with the RMSD and RMSF results (Fig. S3h). A single, deep global low-energy basin (minimum free energy ~0 kJ/mol) was clearly identified, indicating the complex predominantly occupied a single thermodynamically stable dominant conformation throughout the simulation. High-energy unstable states were only sparsely distributed at the landscape edges, confirming the significant energetic advantage of the stable conformation (Fig. S3i). These findings align with previous MD analyses, further verifying the excellent stability of the ligand-protein complex.

**Fig. S3.** Pharmacological analysis of the Flor-MASH network and molecular dynamics simulations of UDCA with FXR. (a) Venn diagram of MASH-associated disease targets; (b) Venn diagram of potential Flor-associated targets; (c) Venn diagram of Database, Drug Target, and RNA-seq data; (d) PPI network of overlapping targets; (e) Heatmap showing the relative mRNA expression (Flor group vs. Mod group); (f) RMSD of the protein, ligand, and protein-ligand; (g) RMSF of the protein-ligand complexes; (h) Hydrogen bond counts in the protein-ligand complexes; (i) 2D interaction diagram of protein–ligand binding modes and Gibbs free energy landscape.

**Fig. S4.** Flor restores the hepatic BA subtype balance disrupted by MASH. (a–g) Hepatic abundances of total BAs (a), 12-OH BAs (b), non-12-OH BAs (c), primary BAs (d), secondary BAs (e), conjugated BAs (f), and unconjugated BAs (g); (h) Compositional profile of 12-OH BAs; (i) Compositional profile of non-12-OH BAs. **P* < 0.05, ***P* < 0.01, ****P* < 0.001, *****P* < 0.0001.

**Table S4.** Summary of GH genes derived from *P. goldsteinii* in the fecal metagenome

| **CAZy_Family** | **Functional_Description** | **E_value** |
| --- | --- | --- |
| GH106 | Glycoside Hydrolases 106 | 2.90E-70 |
| GH78 | Glycoside Hydrolases 78 | 1.80E-103 |
| GH177 | Glycoside Hydrolases 177 | 2.40E-36 |
| GH73 | Glycoside Hydrolases 73 | 9.80E-37 |
| GH163 | Glycoside Hydrolases 163 | 7.80E-104 |
| GH95 | Glycoside Hydrolases 95 | 2.10E-115 |
| GH136 | Glycoside Hydrolases 136 | 8.90E-109 |
| GH42 | Glycoside Hydrolases 42 | 3.50E-124 |
| GH130_2 | Glycoside Hydrolases 130_2 | 1.60E-165 |
| GH43_33 | Glycoside Hydrolases 43_33 | 8.20E-90 |
